# Isotopic niche plasticity of American alligators within the southern Everglades

**DOI:** 10.1371/journal.pone.0326148

**Published:** 2025-06-27

**Authors:** Mathew J. Denton, Michael S. Cherkiss, Frank J. Mazzotti, Laura A. Brandt, Sidney T. Godfrey, Darren Johnson, Kristen M. Hart

**Affiliations:** 1 Wetland and Aquatic Research Center, U.S. Geological Survey, Gainesville, Florida, United States of America; 2 Wetland and Aquatic Research Center, U.S. Geological Survey, Davie, Florida, United States of America; 3 Fort Lauderdale Research and Education Center, University of Florida, Davie, Florida, United States of America; 4 U.S. Fish and Wildlife Service, Davie, Florida, United States of America; 5 Wetland and Aquatic Research Center, U.S. Geological Survey, Lafayette, Louisiana, United States of America; University of Hyogo, JAPAN

## Abstract

Hydrologic alterations within the Everglades have degraded American alligator (*Alligator mississippiensis*) habitat, reduced prey base, and increased physiological stress. Alligator body condition declined across many management areas from 2000 through 2014, prompting us to investigate the relationship between their intraspecific isotopic niche dynamics and body condition. Alligators within the estuary had a larger niche driven by a wider range in stable carbon isotope ratios than those sampled in freshwater habitats. Spatially, model predictability was higher at the smaller scale, reflecting the variability in basal sources and biochemistry among capture sites. Male niches were often larger than those of females, driven by wider ranges of δ^13^C values, suggesting that they differ in their proportional use of habitats and or resources. However, the similar ranges of δ^15^N values indicated both sexes foraged within the same trophic level. Furthermore, while not significantly different, large alligators often had a larger niche with elevated δ^15^N values compared to medium-sized alligators. Although alligators utilize similar stable carbon and nitrogen isotope pools through time, there was considerable temporal variability. These temporal variations in alligators’ isotopic niche were likely influenced by seasonal hydrologic fluctuations within each site, with their niches often being larger in the spring captures than the fall captures. Alligators’ body condition estimates were correlated with intraspecific niche characteristics, including the mean centroid distance between sexes and the interaction between male and female niche size and overlap, within a site, capture period, and year. The variability in intraspecific niche dynamics, landscape heterogeneity, and dynamic hydrology are considerations for designing sustainable management strategies to conserve and enhance alligator populations within the Everglades landscape.

## Introduction

The Everglades is the largest subtropical wetland in the United States [1], and it has been reduced to less than half its original size to support the growing human demand for land and water over the last 100 years. The southern Everglades includes a mosaic wetland made up of freshwater wet prairies, marl prairies, ridge and sloughs, ponds, and tree islands, which are dominated by grasses, shrubs, trees and periphyton [1], transitioning to mangrove forested estuaries and coastlines. Compartmentalization through a system of canals, levees, and roads have modified the landscape and natural water flow, altering hydrology system-wide [2]. For these reasons, the Everglades is currently the focus of one of the world’s largest wetland restoration efforts [3–6]. Evaluating the success of restoration efforts depends on effectively monitoring the ecological indicators that are representative of the system [7]. Monitoring programs such as the Restoration Coordination and Veriﬁcation (RECOVER) [8,9] describes how following trends of, and understanding relationships between, hydrology and ecological indicators are being brought into Everglades restoration planning. RECOVER encompasses multiple watersheds within the southern Everglades including Arthur R. Marshall Loxahatchee National Wildlife Refuge, Water Conservation Areas 2 and 3, Everglades National Park, and Big Cypress National Preserve [10].

American alligators (*Alligator mississippiensis*) cover the entire spatial extent of the Greater Everglades and as a keystone species, are recognized as an indicator species for Everglades ecosystem restoration, with water management having impacts on all life stages [10,11]. Alligators are important in food webs as both predator and prey [12], depending on aquatic and semi-aquatic organisms as food [13,14]. Alligators undergo up to fifteen-fold increases in body size, which coincides with marked changes in diet and habitat use [15,16]. Diet studies have shown alligators to be generalists in both freshwater and estuarine environments, foraging on a variety of prey including fish, crustaceans, gastropods, mammals, reptiles, amphibians, birds, insects, and rays [13–15,17–19]. As they increase in body size, they become more mobile with larger home ranges [20,21], with seasonal variations in their range and habitat preferences [15,22,23]. Males and females’ movement patterns differ seasonally, with reproductively active females often maintaining relatively small home ranges, close to nesting sites [24]; whereas subadult and adult males establish new territories or travel considerable distances to access mates [20,21]. Hydrologic changes in the Everglades (including increases in frequency and intensity of droughts) have degraded habitat, reduced alligator’s prey base, and increased physiological stress [12,25–27]. Body condition indices are used to describe an animal’s health and could be an important determinant of fitness [28]. Alligator body condition is directly linked to the suitability of environmental and hydrologic conditions which vary annually [10,29]. This annual variation is influenced by the interaction of fluctuations in annual, fall and spring water depths [27], which are known to affect the distribution of wetland plant and animal communities [27,30–33]; while alligator distribution, abundance, reproduction, and body condition within mangrove estuaries are controlled by salinity [34]. From the early 2000’s through 2014 alligator body condition had declined across many of the Everglades management areas [27], which may be associated with these fluctuations in available resources potentially influencing their diet and trophic interactions.

Recently stable isotope analysis has increasingly been used to gain insights on various aspects of crocodilian foraging dynamics. Isotopic analysis has been used to identify niche partitioning within a large predator guild in both oligotrophic and tropical freshwater ecosystems [35,36]. Additionally, body size and ontogenetic shifts have been identified in Amazonian crocodilians [37], Nile crocodiles (*Crocodylus niloticus*) [38,39], Saltwater crocodiles (*Crocodylus porosus*) [40] and the American alligator (*Alligator mississippiensis*) [41]. Furthermore, alligator skin samples [42], plasma, and red blood cell (RBC) fractions [14] have been analyzed to investigate habitat linkages between marine and estuarine/freshwater food webs. Stable isotope analyses are commonly used to elucidate patterns in food-webs structure and habitat use [43], as the isotopic composition of the consumer tissues closely tracks the isotopic composition of the consumer’s diet adjusted for changes due to isotopic discrimination during metabolism [44,45]. Stable carbon isotopic ratios in animal tissues, expressed as δ^13^C values, reflect primary producers, and can help differentiate the relative contributions of various plant functional groups with different photosynthetic pathways (e.g., macrophytes versus particulate organic matter, or periphyton versus phytoplankton), and can vary distinctly among habitats (e.g., freshwater versus estuarine/marine) [44–46]. Whereas stable nitrogen isotope ratios (δ^15^N) are used to inform on the trophic level of a species [46,47], and to detect dietary shifts [48]. The development of the isotopic niche, defined as an area (in δ-space) with isotopic values (δ values) as the coordinates [49], is particularly useful for studies of animal interactions in wild populations because the chemical composition of animals (isotope ratio) tends to reflect both the food consumed and the habitats occupied by the species, providing an integrated representation in space and time. For generalists, the size and orientation of the isotopic niche space is inherently plastic, changing in response to available food sources [50–52].

While the isotopic niche offers a means to describe interactions at many ecological levels (e.g., individual, population, community, ecosystem), both intrinsic and extrinsic factors drive isotopic variability and ultimately niche dimensions. Fractionation, routing, and turnover of stable isotopes are influenced by environmental variables (e.g., temperature, rainfall, salinity regimes, etc.) along with the composition of food and consumer, with no two relationships being identical [53–56]. Additionally, animal physiology and behavior can significantly influence isotopic variation both within and between sampled populations based on differences in the size, age, and/or sex of conspecifics. Behavioral differences in home ranges and cross ecosystem foraging vary amongst sexes and size classes potentially exposing them to different resource pools [41], and the rate of isotopic incorporation is influenced by the animal’s body size, growth rate, and metabolic rate of the tissue type sampled [56–61]. Tissues high in lipid content could be ^13^C-depleted relative to their other tissues [44,62], offspring are expected to have lower δ^13^C values and higher δ^15^N values than their mothers [63,64], and increased stress such as from fasting can causing an enrichment in ^15^N produced by catabolism of endogenous proteins [19].

A variety of extrinsic factors may also lead to substantial variability in behavior altering both the ecological (and thus isotopic) niches species fill and their overlap in resource use with other species [65–67]. For example, niche widths may increase or decrease in response to ecosystem changes, with shifts in δ^13^C attributed to changes in the abiotic environment, while shifts in δ^15^N are attributed to changes in the trophic structure of prey communities following habitat conversion (e.g., natural landscapes to agriculture or urban) or fragmentation [52,68]. Thus, as productivity within a habitat increases, a species niche could either increase as they capitalize on an increase in available resources or decrease as it becomes viable to focus on preferred resources. Alternatively, when there is a decrease in productivity, preferred resources may become scarce leading species to increase the breadth of their niche to include additional habitats, or other, less favorable resources. If resources are limiting and lead to different patterns of resource use, it is expected that differences in niche characteristics (e.g., size, shape, overlap) would be observed within and between populations [69–71] and demographic groups [72,73]. Therefore, the spatial extent and temporal variability of a study species and their food sources, can have broad impacts on isotopic niche dynamics and the species associated ecological role [74]. This is likely the case for species within Florida’s Everglades ecosystem with dynamic hydrology and nutrient gradients, affecting patterns of eutrophication and disturbance (i.e., natural cycle of wet/dry seasonal rain delivery, but water level and flow is controlled for anthropogenic needs such as agriculture and flood control).

The recent decline in alligator body condition within the southern Everglades prompted us to investigate their isotopic niches to understand differences in foraging strategies among populations, and how these niches correlate with body condition. The specific objectives of this study were to: (i) evaluate alligators’ δ^13^C and δ^15^N values and isotopic niche amongst the various freshwater wetlands and estuarine river system, (ii) assess the influence of intraspecific groups (sexes or ontogeny) on isotopic niche, and (iii) assess temporal variability in alligators’ isotopic niche, and (iv) assess if intraspecific niche characteristics within alligator populations were correlated with their overall body condition.

## Methods

### Study area

**Fig 1:**
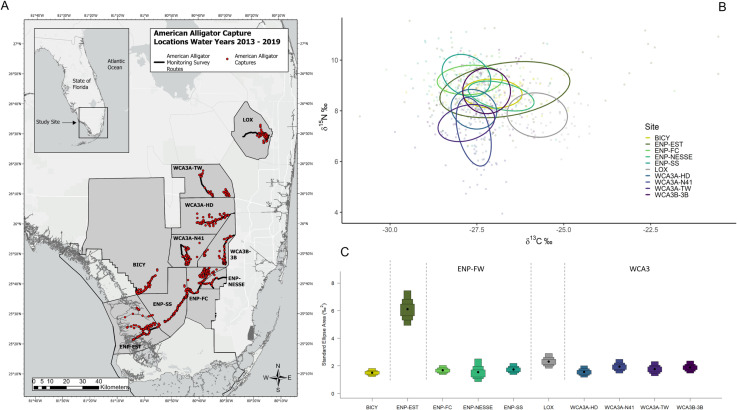
Study sites. **A)** Study sites and American alligator (*Alligator mississippiensis*) capture locations (red dots) within the southern Everglades ecosystem in Florida, USA during water years 2013-2019, their B) estimated 40% core isotopic niche and **C)** Bayesian estimated standard ellipse area (SEA_B_) core niche widths among sties. The ten sampling sites were within five wetland watersheds, Arthur R. Marshall Loxahatchee National Wildlife Refuge (LOX), Big Cypress National Preserve (BICY), estuaries within southwestern Everglades National Park (ENP-EST), and multiple sites within Water Conservation Area 3 (WCA3; WCA3A-TW (Tower), WCA3A-HD (Holiday Park), WCA3A-N41, WCA3B-3B), and Everglades National Park freshwater marshes (ENP-FW; ENP-FC (Frog City), ENP-SS (Shark Slough), ENP-NESSE (NE Shark Slough)).

We utilized samples collected from animals that were captured as part of an ongoing long-term monitoring program within five of the wetlands recognized in RECOVER. Our study sites were within four freshwater wetlands and an estuarine river system within the southern Everglades: Arthur R. Marshall Loxahatchee National Wildlife Refuge (LOX), Water Conservation Area 3 (WCA3), Everglades National Park (ENP), Big Cypress National Preserve (BICY), and the Shark River estuary within ENP (ENP-EST). Due to their compartmentalization, spatial scale, accessibility, varied habitat, and water regimes, we sampled alligators from multiple sampling sites within two of the wetlands. Water Conservation Area 3 included four sites: Tower (WCA3A-TW), Holiday Park (WCA3A-HD), North of Highway 41 (WCA3A-N41), and WCA3B-3B. Everglades National Park included three sites: Frog City (ENP-FC), Shark Slough (ENP-SS), and Northeast Shark Slough East (ENP-NESSE) (Fig 1A). The freshwater wetlands were within the ridge and slough landscape, composed of a mosaic of sloughs, wet prairie, sawgrass marsh, tree islands, periphyton communities, and cattails; while Shark River estuary is a mangrove dominated tidal river with salinity and water depth varying seasonally throughout the estuary between high-precipitation ‘wet’ seasons and low-precipitation ‘dry’ seasons [11,75]. Shark River Slough is the headwater source for Shark River [76], and upstream marshes are dominated by sawgrass (*Cladium jamaicense*) [15]. Hydrology is influenced by rainfall and water management with 75% of rainfall (approximately 1320 mm/year; [1]) occurring May–October (wet season) and 25% November–April (dry season). The subtropical climate of South Florida features hot, humid summers and mild winters, with surface hydrology managed for flood protection, water supply, and ecosystem management. Hydrologic data is reported by water year (Wyear), which begins on May1st of the previous calendar year (marking the start of the wet season) and ends on April 30th of the reporting year (the end of the dry season). Research was conducted under Florida Fish and Wildlife Conservation Commission permit SPGS-17-62 and SPGS-20-60; U.S. Fish and Wildlife Service Arthur R. Marshall Loxahatchee National Wildlife Refuge Special Use Permit B16-003; National Park Service permits BICY-2017-SCI-0004, EVER-2016-SCI-0014, and EVER-2020-SCI-0031.

### Alligator sampling

We captured alligators at night from an airboat or motorboat in both the Spring (February-May) and Fall (September–November) between Wyears 2013−2020. Immediately following capture, we collected ~1.5–2.0 mL of blood via the post-occipital venous sinus or the ventral coccygeal vein with a sterile, 20-gauge 5.0 mL capacity syringe. Between Wyear 2013−2015, we collected plasma (pl) fractions by immediately centrifuging blood in the field using a portable centrifuge, however after Wyear 2015 logistical constraints required us to utilize whole blood (wb). Therefore, in the spring of Wyear 2016 we collected paired plasma and whole blood samples from a subset of alligators to calculate correction factors between the tissues. After collection, we placed plasma fractions and or whole blood samples into individually labeled, sterile Corning Cryovials. We stored the vials on ice in the field until they were transferred into a −20 °C freezer in the laboratory later that same night. All applicable institutional and/or national guidelines for the care and use of animals were followed, and all efforts were made to minimize stress. All field methods were approved by the University of Florida’s Institutional Animal Care and Use Committee (IACUC# 201509072, 201609666, 201809072, and 201909666).

We measured and recorded each alligator’s length measurements (snout-vent length – SVL; and total length – TL) to the nearest 1.0 cm, mass (M) to the nearest 0.1 kg, and noted sex. Each animal was given a unique individual mark and released at the site of capture. We assigned alligators to a size class as follows: small (≥ 50 – < 125 cm TL), medium (≥ 125 – < 175 cm TL), or large (≥ 175 cm TL). Body condition can be measured using simple indices and can provide a rapid assessment of population health [77,78]. We used Fulton’s K (K = M/SVL^3^ × 10^5^) due to its simplicity and historical use evaluating the body condition of alligators within the Everglades [10,27,29,77,78], and we classified body condition as poor (≤1.95), fair (>1.95– ≤ 2.1), good (>2.1– ≤ 2.27), or excellent (>2.27) following Everglades restoration targets [79]. As a result of alligator’s being sampled as part of an ongoing long-term monitoring program assessing abundance and body condition of alligators ≥ 125 cm, it limited the number of small individuals sampled (n = 3) and precluded the inclusion of resource samples.

### Isotope processing

In the laboratory individual samples were thawed and dried at approximately 60 °C for up to 48 hours and then pulverized to a fine homogenized powder using a mortar and pestle. We weighed homogenized plasma and whole blood samples (0.4 mg – 0.6 mg) into 5 x 9 mm pressed tin capsules which were sealed and sent for analysis using standard elemental analyzer isotope ratio mass spectrometer procedures. Samples were analyzed at Florida International University’s (FIU) Stable Isotope Laboratory during calendar years 2015–2017 (131 samples from Wyear 2013(2), 2014(57), 2015(50), 2016(22)), Washington State University’s (WSU) Bioanalytical Laboratory during calendar years 2017–2019 (469 samples from Wyear 2014(43), 2015(46), 2016(20), 2017(231), 2018(2), 2019(127), and University of California, Davis’s (UCD) Stable Isotope Facility during calendar years 2019–2021 (93 samples from Wyear 2014(1), 2015(34), 2016(2), 2017(3), 2019(50), 2020(3).

We compared δ^13^C and δ^15^N values of internal references of bovine liver and duplicated alligator samples from the three labs and detected no statistical difference (α > 0.05), thus no adjustments were made among the three laboratory results prior to analysis. Duplicates of every 10th sample were run to test efficiency of homogenization. Delta values are expressed relative to international standards VPDB (Vienna Pee Dee Belemnite) and Air for carbon and nitrogen, respectively. Results are presented in δ notation (where δ^13^C or δ^15^N = [{R_sample_/R_standard_ −1}] x 1,000, with R = ^13^C/^12^C or ^15^N/^14^N). The standard deviations of internal reference materials used by the laboratories were FIU (glycine, 0.07‰ for δ^13^C and 0.06‰ for δ^15^N), WSU (egg albumin, 0.04‰ for δ^13^C and 0.01‰ for δ^15^N), and UCD (bovine liver and keratin, 0.06‰ for δ^13^C and 0.06‰ for δ^15^N). There was no correlation between C:N and ^13^C among alligator tissue (*P* = 0.9497, t = −0.0631, df = 692), and mean C:N ratios for all alligator tissue in this study was 3.6 ± 0.3SD. In animals with low C:N ratios (aquatic animals <3.5%; terrestrial animals <10%), lipid removal has little effect on δ^13^C values [62]. Therefore, since there was no correlation between C:N and ^13^C, and C:N ratio was low, we did not chemically remove or account for lipids mathematically [62,80,81].

Isotopic ratios of a consumer’s tissues differ from the assimilated resource due to the specific metabolic processes of each tissue. These differences are expressed as diet-tissue discrimination factors (Δ^13^C and Δ^15^N), which influence the resulting isotopic values of a consumer and can lead to misleading inferences when comparing tissues [82]. Thus, to enable us to assess temporal trends in the alligators’ isotopic niches using different tissues, we first calculated variable fractionation factors between the two tissue types [82]. We used linear regression equations derived from 28 paired tissues (plasma and whole blood) [spring of Wyear 2016: 27 alligators of both sexes (1 medium female: TL: 172.6 cm; and 25 large: 185.1–313.8 cm 10 female, 17 male), from multiple body condition categories, and from three sites (BICY [14], ENP-FC [7], ENP-SS [6]); and one male from Wyear 2014 (ENP-SS 270 cm TL)]. Plasma values were highly correlated with whole blood values for both δ^13^C (r = 0.92699) and δ^15^N (r = 0.82962), with >99% power for both adjustment calculations. There were less plasma samples (237) than whole blood (456), thus, to account for the isotopic differences between the tissues we applied the calculated fractionation to the δ values of the plasma tissues collected from Wyears 2014–2016 to obtain the adjusted values (adj_ δ values) for the table which were used in the analyses. Differential δ^13^C adjustment values were necessary for males versus females [Males: adj_C^13^ = (2.64268 + 1.08907 * C^13^pl); ~ +0.22‰, SD = 0.63/ Females: adj_C^13^ = (−11.04367 + 0.58652 * C^13^pl); ~ +0.40‰, SD = 0.48], while the same δ^15^N adjustment value was applied for both sexes (adj_N^15^ = (0.6150 + 1.1166 * N^15^pl); ~ +1.49‰, SD = 0.55; S1A and S1B Fig illustrates niche adjustments). Isotopic turnover, expressed as isotopic half-life, is the time (in days) required for 50% equilibration with a new diet. Previous studies on ectotherm turnover rates (alligators, caiman, sea turtles, and pond turtles) showed similarities among tissues with turnover rates influenced by metabolic activity (plasma < muscle/WB < liver < RBC) [57,59,60,83]. The rate at which isotope values from resources are incorporated into alligator tissues is relatively slow compared to endothermic species [60], with plasma half-life around 62 days for both δ^13^C and δ^15^N, and red blood cells around 141 and 277 days respectively. Although alligator whole blood was not analyzed as part of that study [60], based on previous studies it is reasonable to assume the turnover rates are somewhere between the two, suggesting half-lives between 100–120 days for δ^13^C and 120–180 days for δ^15^N (See S1C Fig for estimated timeline).

### Statistical analysis

We analyzed data from 693 sampling events (n_Plasma_ = 237, n_WholeBlood_ = 456) from 652 individual alligators, with 37 alligators captured and sampled twice (19 Males [large: 18; medium: 1], 18 Females [large: 15; medium: 3]), and two alligators (1 male and 1 female) sampled three times. There were 290 females (large: 234; medium: 55; small: 1), 357 males (large: 320; medium: 35; small: 2), and five individuals of unknown sex (medium: 5). Of the 693 samples, 678 were included in our isotopic modeling as we excluded alligators with an undetermined sex (n = 5), and when variables contained less than five samples [WYs- 2013 (n = 2), 2018 (n = 2), 2020 (n = 3), size class: small (n = 3)]. For all analyses, the variable “tissue type” was not used in the analyses because it was confounded with water year (2013–2016: plasma and 2017–2020: whole blood). To investigate the isotopic variance of alligators across the Everglades we first compared alligators’ bivariate stable carbon and nitrogen isotope ratios among sites (or wetlands), sexes, size classes (sclass), capture period (capperiod), and Wyear via generalized linear models, multiple analysis of variance (MANOVA), and Akaike Information Criterion model selection for small sample sizes (AICc). We used an α = 0.05 for all statistical tests. A type IV sums of squares was fit to the generalized linear model to adjust for the missing treatment combinations (e.g., Wyear*site interaction). The residuals of stable carbon and nitrogen ratios were correlated and a MANOVA was run (p-value = 0.0012). We assessed AICc modeling results to determine the top model for each response. Model residuals were checked for each response to test the assumptions of homogeneity and normality via residual plots. A Tukey’s test was used for mean comparisons of the main univariate effects and a Bonferroni correction (α/number of comparisons) was used for any crossed and nested effects.

### Isotopic niche

Once we evaluated the mean isotopic ratios among groups, to compare alligators’ intraspecific niches we calculated the standard ellipse areas corrected for small sample size (SEA_C_) via maximum likelihood using the variance and covariance of bivariate isotope data using the package ‘SIBER’ v2.1.6 [84] in R statistical software v.4.1.2 [85]. To represent the mean core isotopic niche, we used standard ellipses which contained approximately 40% of bivariate isotope data [84,86]. We used the ellipse SEA_C_ to calculate the degree of isotopic niche overlap, representing a quantitative measure of diet similarity and partitioning of resource and habitat use among groups. We calculated the overlap between sclass, sex, and the sex*sclass interaction (e.g., Large: Male vs. Female or Female: Large vs. Medium, etc.) for Wyear, site, and capture period combinations. We calculated the horizontal distance (xdist; δ^13^C) and vertical distance (ydist; δ^15^N) between the centroids as well as calculating the Euclidean distance (length of the line segment between the centroids) using PROC DISTANCE in SAS 9.4 for each of the sex, sclass, and sex*sclass interactions related to the overlap comparisons above. Ellipse figures were created using the stat_ellipse function within the ‘ggplot2’ package [87].

We then used the estimated Bayesian standard ellipse area metrics (SEA_B_) from package ‘SIBER’ v2.1.6 [84] for niche width comparisons among groups to assess the influence of site, sex, size class, capture period, and Wyear. Bayesian standard ellipse areas were calculated through Markov chain Monte Carlo simulations with 10^4^ iterations, 10^3^ burn-ins, and two chains [84] and the median and 50%, 75%, and 95% credible intervals were reported. Next, we compared areas among groups using pairwise tests using SEA_B_ values drawn in the simulations for each group and calculating the probability that one group was larger (reference group) than the other: SEA_B–groupA _> SEA_B–groupB_ [84].

### Isotopic characteristics and body condition

To detect if there was a pattern between body condition and δ^13^C and δ^15^N values we first ran a MANOVA with on site, Wyear, sex, sclass, and body condition. The errors were correlated, but normality did not hold. For the univariate analyses, both δ^13^C and δ^15^N had normality for Wyear = 2016 and 2019 only. Using AICc model comparisons, the model for both δ^13^C and δ^15^N were a non-significant body condition effect. Making body condition categorical did not change this result. Thus, we investigated the effects of body condition on δ^13^C and δ^15^N values and found no significant effects at either level (wetland: S2A and S2B Fig; site: S2C and S2D Fig).

After determining that body condition was not correlated with either δ^13^C or δ^15^N values, to compare relationship between body condition and isotopic niche we ran an AICc regression model comparison on the mean body condition (mbcond) and predictors: site, sex, sclass, capture period, Wyear, centroid distance (1: vertical[ydist] and horizontal distance[xdist]; or 2: Euclidean distance = sqrt[(xdist^2 + ydist^2)), ellipse overlap (overlap), and the SEA_C_. We also added an interaction with overlap for each variable. We then used the R software package ‘AICcmodavg’ to find the best regression model with the lowest AICc for any model (non-hierarchy, nonlinear regression) and a hierarchy model (which requires that all terms in the interaction occur as main effects). We tested the residuals for homogeneity and normality via residual plots. We applied a Bonferroni correction [α/(number of comparison)] when appropriate, and used PROC GLM in SAS 9.4 to get the final body condition regression estimates. Due to sample size issues (i.e., the small number of medium alligators each capture period and Wyear) only the following models were run: 1) Wyear, site, and capture period, overlaps of the sexes (large alligators only); 2) Wyear, site and capture period, centroid distance (Euclidean), and overlaps of the sexes (medium and large alligators); and 3) Wyear, site and capture period, overlaps of the sclass. Niche overlap was estimated using the maxLikOverlap function in R, PROC GLM in SAS 9.4 was used to derive the final body condition regression estimates. All data analyzed during this study are included as a U.S. Geological data release [88].

## Results

### Factors influencing δ^13^C and δ^15^N values

Alligators in the estuary exhibited a wider range of δ^13^C values than those in any freshwater wetland, while δ^15^N values were highest within ENP-FW and lowest at LOX (Table 1, Fig 1B). Alligators’ isotopic ratios among sites in WCA3 and ENP-FW were more similar than those between the two wetland areas (S1 Table). MANOVA indicated the average bivariate response of δ^13^C and δ^15^N is affected by the following terms: sclass, sex, sex*site, Wyear*site, Wyear* capperiod, site* capperiod, and capperiod *site*Wyear. Wetland was also significant; however, site was more predictive due to the variation between sites within ENP-FW and WCA3 and thus was used for all subsequent analyses. The highest order significant interactions are sclass, sex*site, and capperiod*site*Wyear. Sex, size class, and temporal correlations are discussed in supplementals (S1 Appendix).

**Table 1:** Alligators’ mean isotopic and body condition values.

Site Water Year	Female						Male						Large								Medium				
	(n)	δ^13^C	SD	δ^15^N	SD	Fulton’s K	(n)	δ^13^C	SD	δ^15^N	SD	Fulton’s K	(n)	δ^13^C	SD	δ^15^N	SD	Fulton’s K	(n)	δ^13^C	SD	δ^15^N	SD	Fulton’s K
**BICY (2014–2017)**	**24**	**−27.1**	**0.6**	**8.5**	**0.5**	**2.25**	**76**	**−27.0**	**0.9**	**8.7**	**0.6**	**2.12**	**98**	**−27.1**	**0.8**	**8.7**	**0.6**	**2.15**	**2**	**−26.1**	**0.1**	**8.1**	**0.1**	**2.06**
2014	6	−26.9	0.6	8.6	0.6	2.13	21	−26.6	0.7	8.8	0.5	2.02	25	−26.7	0.7	8.8	0.5	2.0	2	−26.1	0.1	8.1	0.1	2.1
2015	8	−27.1	0.2	8.3	0.5	2.36	20	−27.2	1.1	8.6	0.6	2.14	28	−27.2	0.9	8.5	0.6	2.2	.	.	.	.	.	.
2016	4	−27.7	1.0	8.3	0.2	2.33	10	−27.3	0.7	8.3	0.7	2.22	14	−27.4	0.8	8.3	0.6	2.3	.	.	.	.	.	.
2017	6	−26.8	0.4	8.9	0.3	2.17	25	−27.2	0.9	9.0	0.5	2.14	31	−27.2	0.8	9.0	0.4	2.1	.	.	.	.	.	.
**ENP-EST (2013–2020)**	**11**	**−27.0**	**1.5**	**8.1**	**0.8**	**2.10**	**85**	**−26.7**	**1.8**	**8.9**	**1.1**	**2.21**	**90**	**−26.7**	**1.9**	**8.9**	**1.1**	**2.20**	**6**	**−27.1**	**0.6**	**8.1**	**0.8**	**2.13**
2013	.	.	.	.	.	.	2	−26.4	0.4	9.1	0.5	2.40	2	−26.4	0.4	9.1	0.5	2.4	.	.	.	.	.	.
2014	3	−26.7	0.8	8.1	0.6	2.13	19	−26.0	2.1	9.2	1.1	2.10	21	−26.0	2.0	9.1	1.1	2.1	1	−27.5	.	8.3	.	2.3
2015	5	−27.5	1.0	8.0	0.9	2.21	22	−27.3	1.6	9.5	1.1	2.37	24	−27.4	1.6	9.4	1.2	2.4	3	−27.0	0.8	8.0	1.3	2.2
2016	.	.	.	.	.	.	10	−27.6	1.3	8.4	0.8	2.23	8	−27.7	1.4	8.6	0.9	2.3	2	−27.2	0.2	8.0	0.1	2.0
2017	2	−25.9	3.7	8.8	0.4	1.72	24	−26.2	2.1	8.8	0.8	2.14	26	−26.2	2.1	8.8	0.7	2.1	.	.	.	.	.	.
2019	1	−27.3	.	6.8	.	2.15	5	−26.6	0.5	7.2	0.3	2.34	6	−26.7	0.5	7.2	0.3	2.3	.	.	.	.	.	.
2020	.	.	.	.	.	.	3	−26.6	0.7	8.6	0.3	1.96	3	−26.6	0.7	8.6	0.3	2.0	.	.	.	.	.	.
**ENP-FC (2014–2019)**	**51**	**−27.9**	**0.6**	**9.2**	**0.6**	**2.12**	**48**	**−27.5**	**1.2**	**9.2**	**0.6**	**2.10**	**87**	**−27.7**	**0.9**	**9.3**	**0.6**	**2.13**	**12**	**−27.8**	**0.9**	**8.8**	**0.4**	**2.02**
2014	9	−27.8	0.3	9.4	0.7	2.05	7	−27.0	2.3	9.2	0.6	2.26	15	−27.4	1.6	9.3	0.7	2.1	1	−28.0	.	9.2	.	2.1
2015	12	−27.8	0.6	9.3	0.5	2.29	10	−27.2	1.0	9.5	0.4	2.11	20	−27.4	0.8	9.5	0.4	2.2	2	−28.1	1.1	8.8	0.0	2.2
2016	5	−27.8	0.2	9.6	0.4	2.02	3	−27.3	0.1	9.1	1.1	2.07	8	−27.6	0.3	9.4	0.7	2.0	.	.	.	.	.	.
2017	15	−27.7	0.8	9.2	0.5	2.03	15	−27.7	0.7	9.4	0.6	2.03	27	−27.7	0.8	9.3	0.6	2.0	3	−27.6	0.4	9.2	0.4	1.9
2019	10	−28.4	0.4	8.9	0.5	2.18	13	−27.9	0.8	8.8	0.4	2.10	17	−28.2	0.4	9.0	0.4	2.2	6	−27.8	1.2	8.5	0.2	2.0
**ENP-NESSE (2019)**	**3**	**−26.3**	**1.1**	**8.0**	**0.9**	**2.25**	**12**	**−26.9**	**0.9**	**8.7**	**0.3**	**2.17**	**12**	**−26.9**	**0.9**	**8.8**	**0.3**	**2.22**	**3**	**−26.3**	**1.1**	**7.9**	**0.9**	**2.07**
2019	3	−26.3	1.1	8.0	0.9	2.25	12	−26.9	0.9	8.7	0.3	2.17	12	−26.9	0.9	8.8	0.3	2.2	3	−26.3	1.1	7.9	0.9	2.1
**ENP-SS (2014–2019)**	**36**	**−27.7**	**0.6**	**9.2**	**0.9**	**2.00**	**35**	**−27.9**	**0.8**	**9.6**	**0.7**	**2.02**	**68**	**−27.8**	**0.7**	**9.4**	**0.8**	**2.01**	**3**	**−28.2**	**1.1**	**8.8**	**0.5**	**1.95**
2014	11	−27.9	0.6	9.6	0.6	1.94	7	−27.9	1.3	10.4	0.7	1.98	17	−27.9	0.9	10.0	0.7	2.0	1	−27.9	.	8.7	.	1.9
2015	11	−27.8	0.4	9.1	1.2	2.05	9	−28.2	0.7	9.6	0.4	2.11	19	−27.9	0.5	9.3	1.0	2.1	1	−29.4	.	9.3	.	1.9
2016	4	−27.7	0.3	9.3	0.5	2.08	8	−27.8	0.6	9.7	0.4	1.98	12	−27.8	0.5	9.5	0.5	2.0	.	.	.	.	.	.
2017	8	−27.2	0.3	8.6	0.6	1.98	7	−27.4	0.3	9.5	0.6	1.92	14	−27.3	0.4	9.1	0.7	1.9	1	−27.2	.	8.3	.	2.0
2019	2	−28.5	0.2	8.8	0.4	2.05	4	−28.4	0.3	8.6	0.5	2.12	6	−28.4	0.3	8.6	0.4	2.1	.	.	.	.	.	.
**LOX (2014–2019)**	**50**	**−26.0**	**0.8**	**7.8**	**0.8**	**2.09**	**23**	**−25.3**	**0.8**	**7.9**	**1.0**	**2.02**	**45**	**−25.8**	**0.9**	**8.0**	**0.9**	**2.05**	**30**	**−25.9**	**0.9**	**7.5**	**0.7**	**2.14**
2014	10	−26.6	0.5	8.3	0.7	1.94	3	−25.5	0.6	8.1	0.7	1.78	7	−26.3	0.5	8.6	0.6	1.9	6	−26.3	0.9	7.9	0.7	1.9
2015	15	−26.1	0.5	7.9	1.0	2.09	5	−24.8	0.5	7.8	0.6	1.86	9	−25.9	0.9	8.1	1.0	1.9	11	−25.7	0.6	7.6	0.8	2.1
2017	15	−26.0	0.9	7.8	0.5	2.19	11	−25.5	0.9	8.1	1.2	2.13	20	−25.8	0.9	8.1	0.9	2.1	9	−26.3	0.9	7.3	0.3	2.3
2019	10	−25.1	0.7	7.1	0.5	2.10	4	−25.0	0.6	7.0	0.3	2.09	9	−25.2	0.7	7.1	0.4	2.1	4	−24.7	0.4	6.9	0.6	2.1
**WCA3A-HD (2017 & 2019)**	**33**	**−27.8**	**0.7**	**8.1**	**0.7**	**2.14**	**23**	**−27.3**	**0.6**	**8.0**	**0.8**	**2.12**	**50**	**−27.6**	**0.7**	**8.1**	**0.8**	**2.13**	**7**	**−27.1**	**0.5**	**7.8**	**0.8**	**2.09**
2017	16	−27.6	0.7	8.6	0.6	2.16	11	−27.1	0.6	8.6	0.5	2.19	23	−27.4	0.7	8.6	0.6	2.2	5	−27.0	0.6	8.3	0.4	2.1
2019	17	−27.9	0.6	7.7	0.6	2.12	12	−27.5	0.5	7.4	0.5	2.05	27	−27.8	0.6	7.6	0.6	2.1	2	−27.2	0.0	6.8	0.5	2.1
**WCA3A-N41 (2015–2019)**	**38**	**−27.7**	**0.4**	**7.1**	**1.4**	**2.16**	**22**	**−27.4**	**0.5**	**7.3**	**1.2**	**2.08**	**44**	**−27.5**	**0.5**	**7.0**	**1.4**	**2.16**	**16**	**−27.9**	**0.4**	**7.6**	**1.1**	**2.04**
2015	4	−27.5	0.3	7.7	0.3	2.10	1	−27.0	.	8.0	.	1.94	4	−27.4	0.4	7.8	0.4	2.0	1	−27.4	.	7.7	.	2.2
2017	17	−28.0	0.4	8.3	0.5	2.06	11	−27.5	0.7	8.3	0.6	1.98	16	−27.6	0.6	8.4	0.6	2.0	12	−28.0	0.4	8.2	0.4	2.0
2019	17	−27.5	0.4	5.8	1.0	2.27	10	−27.3	0.3	6.1	0.6	2.21	24	−27.4	0.4	6.0	0.9	2.3	3	−27.5	0.3	5.5	0.2	2.1
**WCA3A-TW (2017–2019)**	**25**	**−27.7**	**0.8**	**7.7**	**0.7**	**2.24**	**22**	**−27.9**	**0.8**	**7.3**	**0.7**	**2.27**	**37**	**−27.8**	**0.8**	**7.6**	**0.8**	**2.29**	**10**	**−27.8**	**0.9**	**7.4**	**0.5**	**2.12**
2017	11	−27.3	0.8	8.3	0.5	2.15	4	−26.9	0.3	7.9	0.4	2.12	11	−27.3	0.8	8.4	0.3	2.2	4	−26.9	0.4	7.6	0.4	2.0
2018	1	−27.7	.	7.3	.	2.13	1	−28.1	.	7.7	.	2.12	2	−27.9	0.2	7.5	0.3	2.1	.	.	.	.	.	.
2019	13	−28.1	0.7	7.2	0.3	2.32	17	−28.1	0.7	7.2	0.7	2.31	24	−28.1	0.6	7.2	0.6	2.3	6	−28.3	0.7	7.2	0.5	2.2
**WCA3B-3B (2014–2019)**	**39**	**−27.4**	**0.7**	**8.8**	**0.9**	**2.13**	**32**	**−27.1**	**0.7**	**8.8**	**0.9**	**2.16**	**60**	**−27.2**	**0.7**	**8.8**	**0.9**	**2.14**	**10**	**−27.3**	**0.4**	**8.6**	**0.9**	**2.16**
2014	4	−27.1	0.6	9.8	0.8	2.00	1	−27.7	.	10.2	.	1.92	5	−27.2	0.6	9.9	0.7	2.0	.	.	.	.	.	.
2015	6	−27.2	0.7	9.6	0.4	1.82	2	−26.9	0.8	9.6	0.4	1.74	5	−27.0	0.7	9.5	0.4	1.6	3	−27.4	0.6	9.6	0.2	2.1
2017	10	−27.4	0.8	9.2	0.6	2.21	21	−27.1	0.7	9.0	0.8	2.22	27	−27.2	0.8	9.2	0.7	2.2	3	−27.2	0.1	8.5	0.8	2.3
2019	19	−27.5	0.6	8.0	0.4	2.22	8	−26.9	0.6	7.8	0.6	2.12	23	−27.3	0.7	8.0	0.4	2.2	4	−27.3	0.4	7.9	0.6	2.2

Mean and SD of δ^13^C and δ^15^N values, Fulton’s K [K = M/SVL^3^ × 10^5^] and associated body condition [Poor (≤1.95), Fair (>1.95– ≤ 2.1), Good (>2.1– ≤ 2.27), or Excellent (>2.27)] from the 693 American alligator (*Alligator mississippiensis*) tissue samples within the southern Everglades between Wyear 2014−2019. The ten sampling sites were within five wetland watersheds, Arthur R. Marshall Loxahatchee National Wildlife Refuge (LOX), Big Cypress National Preserve (BICY), estuaries within southwestern Everglades National Park (ENP-EST), and multiple sites within Water Conservation Area 3 (WCA3; WCA3A-TW (Tower), WCA3A-HD (Holiday Park), WCA3A-N41, WCA3B-3B), and Everglades National Park freshwater marshes (ENP-FW; ENP-FC (Frog City), ENP-SS (Shark Slough), ENP-NESSE (NE Shark Slough).Values from 2014−2015 represent adjusted plasma values, unadjusted δ^13^C and δ^15^N values can be found in the associated data release [88]. (* Values of Males and Females exclude five individuals when sex was undetermined in 2017 [LOX: n = 4, mean δ^13^C −26.7 SD 0.8, δ^15^N 7.1 SD 0.1, body condition 2.32; WCA3-HD: n = 1, mean δ^13^C −26.2, δ^15^N 7.9, body condition 1.87], and values of Size classes excludes three small alligators [LOX 2017: δ^13^C −24.6, δ^15^N 7.1, Fulton’s K: 2.07, 2019: δ^13^C −25.1, δ^15^N 7.1, Fulton’s K: 2.11; and 2017 WCA3B-3B: δ^13^C −27.7, δ^15^N 7.9, Fulton’s K: 2.16]).

### Isotopic niche

**Fig 2:**
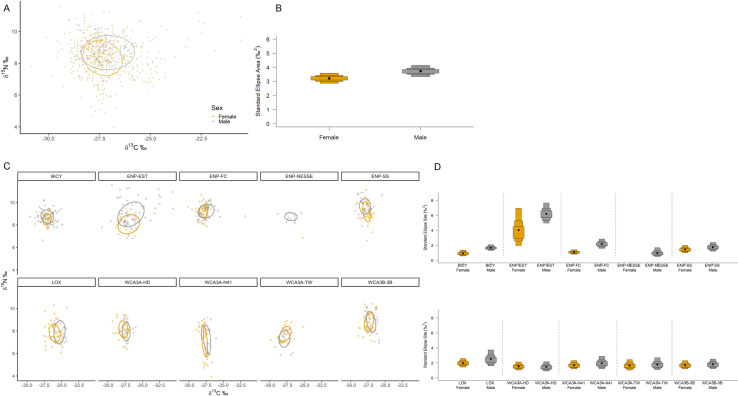
Alligators’ core isotopic niche between sexes. American alligators (*Alligator mississippiensis*) estimated isotopic niches and corresponding Bayesian estimated ellipse area (SEA_B_) niche widths for each sex within all study sites combined (A, B) and within each study site (C, D) within the southern Florida Everglades, USA. Ellipses represent the 40% core isotopic niche space. Boxes represent 50%, 75%, and 95% credibility intervals, and black dots correspond to the median.

**Fig 3:**
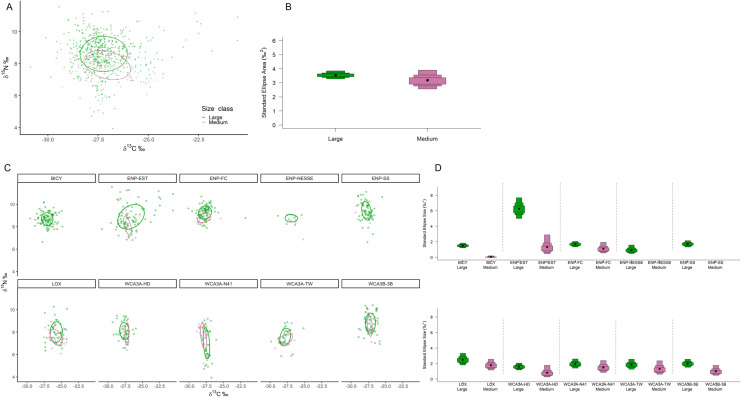
Alligators’ core isotopic niche between size classes. American alligators (*Alligator mississippiensis*) estimated isotopic niches and corresponding Bayesian estimated ellipse area (SEA_B_) niche widths by size class within all study sites combined (A, B) and within each study site (C, D) within the southern Florida Everglades, USA. Alligators with a total length (TL) > 1.25 cm and <1.75 cm were classified as medium, those with TL ≥ 1.75 cm were classified as large. Ellipses represent the 40% core isotopic niche space. Boxes represent 50%, 75%, and 95% credibility intervals, and black dots correspond to the median.

**Fig 4:**
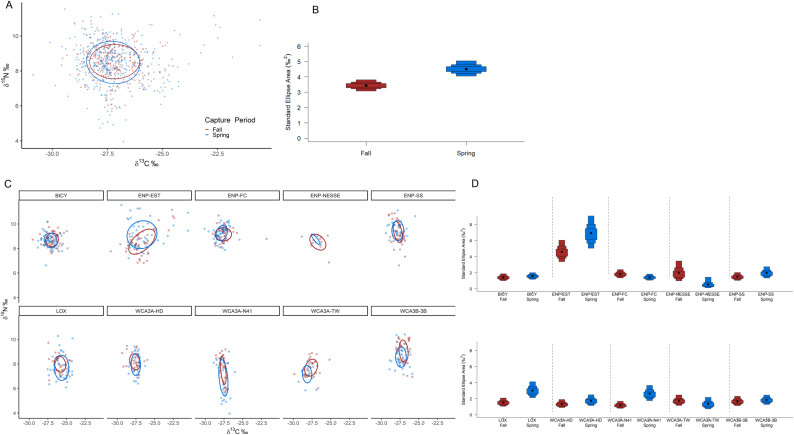
Alligators’ core isotopic niche between capture periods. American alligators (*Alligator mississippiensis*) estimated isotopic niches and corresponding Bayesian estimated ellipse area (SEA_B_) niche widths during each capture period (Spring: Feb-May; Fall: September-November) within all study sites combined (A, B) and within each study site (C, D) within the southern Florida Everglades, USA. Ellipses represent the 40% core isotopic niche space. Boxes represent 50%, 75%, and 95% credibility intervals, and black dots correspond to the median.

**Fig 5:**
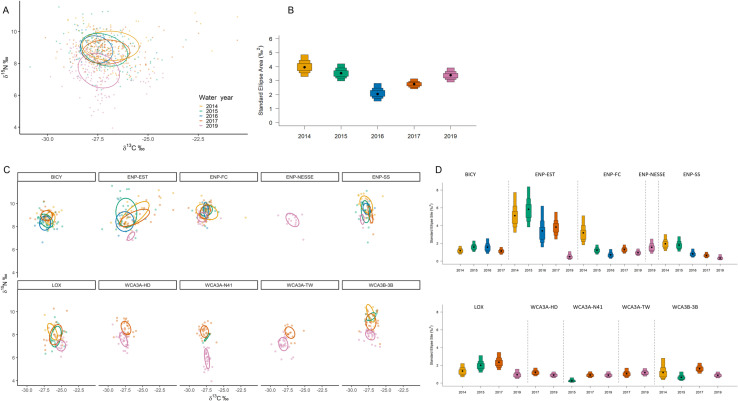
Alligators’ core isotopic niche between water years. American alligators (*Alligator mississippiensis*) estimated isotopic niches and corresponding Bayesian estimated ellipse area (SEA_B_) niche widths during each water year (Wyear; May 1 of the previous year to April 30 of the reporting year) within all study sites combined (A, B) and within each study site (C, D) within the southern Florida Everglades, USA. Ellipses represent the 40% core isotopic niche space. Boxes represent 50%, 75%, and 95% credibility intervals, and black dots correspond to the median. Only 4 sites were sampled in 2016.

SIBER analysis revealed overall niche of alligators from within the estuary were significantly larger than those from the interior freshwater wetland sites, which were more similar in size (Fig 1B and 1C). Of the wetlands with multiple capture sites (WCA3 and ENP-FW), there was more variation between wetlands than among sites, where they differed more in their position in iso-space than niche size (Fig 1B and 1C, S3 Fig). All sites combined, males exhibited a slightly larger niche than females, however Maximum Likelihood estimates indicate both male and female alligators occupy similar niche sizes within their respective wetland/site (Fig 2). Large alligators occupied larger niches than medium alligators, with the greatest niche differences exhibited by alligators within ENP-EST (Fig 3). The overlap in niche between sexes and size classes varied among capture period (season) and water year within each wetland/site (S4A and S4B Fig). Alligators’ combined niche was greater in the spring capture period than fall (Fig 4A and 4B), however within ENP-FC, ENP-NESSE, WCA3A-TW, and WCA3B-3B alligators’ niche was larger during fall captures (Fig 4C and 4D). There was significant variation in alligators’ niche widths and/or locations in iso-space among years within each site, with ENP-EST and sites within WCA3 exhibiting the greatest variability (Fig 5).

### Body condition analyses using niche (SEA) as a predictor

Of the 678 alligator samples included in the analysis, ~ 27% of the alligators were in excellent condition, ~ 26% good, ~ 24% fair, and ~22% were in poor condition. The body condition of both sexes was often similar, showing no significant difference (p = 0.925), following similar patterns based on wetland, site, and water year (Table 1, S3 Table, S5A and S5B Fig). Results of Model 1 showed the overall model was significant (p-value = 0.0003, R-square = 0.8076), and within this model the variables Site (p-value = 0.0060) and Wyear (p-value = 0.0022) were significant (Table 2). The intercept of the regression model is affected (higher or lower) based on what site and Wyear it is in, but there were no significant slope effects. However, this only represented large-sized alligators and is not directly applicable to real world situations as it excludes the medium size class, thus we performed and focused on Model 2 which included both size classes. For Model 2, the overall model was significant (p-value<0.0001, R-square = 0.7320), and within this model the variables Sex (p-value = 0.0335), site (p-value<0.0001), capperiod (p-value = 0.0021), distance (Euclidean) (p-value = 0.0070), and SEA_C_ *overlap (p-value = 0.0128) were significant. The body condition rate changes based on the distance as well as the SEA_C_ *overlap interaction. As distance increases or decreases, the body condition rate increases or decreases. As both the SEA_C_ and overlap increases or decreases, the body condition rate increases or decreases. When one increases and the other decreases, the body condition changes by the multiplicative factor of SEA_C_ *overlap. Given the site, sex, and capperiod, the intercept of the regression model changes. The slope coefficient for distance gives the rate of change of body condition over distance. Likewise, the SEA_C_ *overlap coefficient gives the change of body condition over SEA_C_ and overlap. The intercept of the regression model is affected (higher or lower) based on what sex, site, and capperiod it is in. Model 3 was not significant whether using Euclidean distance or xdist and ydist in the model. Table 2 shows regression coefficients for significant models only.

**Table 2:** Alligators’ body condition regression coefficients for Models 1 and Model 2.

Model 1						Model 2					
Parameter	Estimate		Standard	t Value	Pr > |t|	Parameter	Estimate		Standard	t Value	Pr > |t|
			Error						Error		
**Intercept**	2.342547	B	0.045729	51.23	<.0001	**Intercept**	2.256059	B	0.068942	32.72	<.0001
**Site 3B**	−0.260051	B	0.091457	−2.84	0.0117	**Sex Female**	0.050357	B	0.022333	2.25	0.0335
**Site BICY**	−0.327599	B	0.087364	−3.75	0.0017	**Sex Male**	<0.0001	B	.	.	.
**Site FC**	−0.417130	B	0.084319	−4.95	0.0001	**Site 3B**	−0.187374	B	0.058869	−3.18	0.004
**Site HD**	−0.265844	B	0.064670	−4.11	0.0008	**Site BICY**	−0.125152	B	0.058560	−2.14	0.043
**Site LOX**	−0.322891	B	0.091457	−3.53	0.0028	**Site FC**	−0.428356	B	0.074898	−5.72	<.0001
**Site N41**	−0.082503	B	0.064670	−1.28	0.2203	**Site HD**	−0.343541	B	0.069376	−4.95	<.0001
**Site SS**	−0.512573	B	0.087364	−5.87	<.0001	**Site LOX**	−0.081298	B	0.070803	−1.15	0.2622
**Site TW**	0.000000	B	.	.	.	**Site N41**	−0.331517	B	0.061395	−5.4	<.0001
**Wyear 2015**	0.258443	B	0.073735	3.51	0.0029	**Site SS**	−0.435997	B	0.071368	−6.11	<.0001
**Wyear 2017**	0.122180	B	0.064670	1.89	0.0771	**Site TW**	0.000000	B	.	.	.
**Wyear 2019**	0.000000	B	.	.	.	**season Fall**	−0.109883	B	0.031800	−3.46	0.0021
						**season Spring**	<0.0000	B	.	.	.
						**distance**	0.233997		0.079264	2.95	0.007
						**SEA**_**C**_ *overlap	0.200084		0.074348	2.69	0.0128

Model 1: (Large alligators only) Intercept is affected by Wyear and site.

‘Mean body condition’ = 2.34254 + ‘site intercept effect’ + ‘wyear intercept effect’. (e.g., for site=’3B’ and Wyear = 2015, ‘Mean body condition’ = 2.34254–0.260051 + 0.258443 = 2.340932).

Model 2: (Large and medium alligators) Intercept is affected by sex, site, season. Slope is affected by distance and SEA_C_*overlap. Distance = the Euclidean distance between centroids.

‘Mean body condition’ = 2.25605 + ‘site intercept effect’ + ‘sex intercept effect’ + ‘season intercept effect’ + 0.23399*distance + 0.20008* SEA_C_ *overlap.

Parameter gives the variable in the model; the estimate gives the coefficient.

## Discussion

### Influence of wetlands on alligators’ niche characteristics

**Fig 6:**
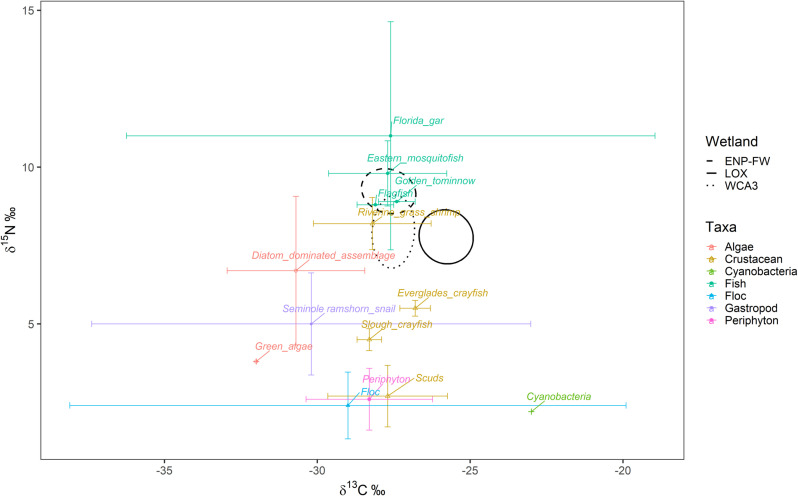
Bi plot of American alligators and potential resources within the southern Everglades. A bivariate isotope plot of δ^13^C and δ^15^N values of taxa (mean ± SD) within the Loxahatchee National Wildlife Refuge (LOX), Water Conservation Area 3 (WCA3), and Everglades National Park (ENP; Shark River Slough and Taylor Slough) in Florida, USA (Isotopic data from taxa other than alligators was adapted from previous Everglades research sampled September 2002 and February 2003 [30], and November-December 2010 [31]). Ellipses represent the 40% core isotopic niche of American alligators (*Alligator mississippiensis*) sampled during water years 2014-2019.

There is considerable spatial variability in nutrient sources and biogeochemical process across the Everglades, with each site having its own unique influence of local biogeochemistry. In the areas we sampled, previous studies have reported similar variations in the δ^13^C and δ^15^N values of vegetation [89,90] and primary consumers [30,31,91]. In each of those studies, baseline values for primary producers and fauna differed among wetlands [30,31], which suggests that the variations in alligators’ isotopic values and their position in δ-space we observed, reflect these baseline differences rather than shifts in trophic levels among sites. Although not directly comparable due to spatiotemporal fluctuations in baseline levels, our study found that alligators had lower δ^15^N values than previously reported for small and medium-sized fish (mosquito fish, gar), instead aligning more closely with values for scuds, snails, grass shrimp, and crayfish within their respective habitats [30,31] (Fig 6). While baseline nitrogen levels have likely shifted, it is common for alligators to have lower δ¹⁵N values than some predatory fish or water snakes, as their generalist diets often include lower trophic prey (e.g., crustaceans, snails, and pond apple) with lower nitrogen values [14,17]. Estuarine alligators occupied the widest range of δ^13^C (−20.56 to −31.57‰) indicating they utilize both estuarine and freshwater resources. However, their range of δ^15^N values were similar to those from freshwater wetlands, suggesting foraging at comparable trophic levels. While WCA3 had largest range in δ^15^N (3.95 to 10.70‰), this was primarily due to a drop in values in 2019, otherwise δ^15^N ranged from (5.14 to 10.7‰) across all sites. Although alligators’ isotopic values and niches within the four FW wetlands (LOX, WCA3A, BICY, ENP-FW) were similar in range and size, they varied in geometry, position, and temporal changes likely based on the specific habitat characteristics, environmental conditions, and prey availability (Fig 3A and 3B). Of the two wetlands with multiple sites (WCA3 and ENP-FW), alligators within ENP-FW sites showed less variation than those in WCA3 (Fig 4A). This difference is likely due to the greater distance and higher temporal variation between capture locations within WCA3, where there are fewer restrictions on search areas within water conservation areas. Additionally, the compartmentalization from levees and canals creates distinct water levels, biochemistry, and basal communities within each site. In contrast, captures in ENP-FW are limited to within 1 kilometer of the trail, resulting in more consistent search areas. Furthermore, restoration efforts have aimed to improve the connectivity through increased and naturally timed water flow, thereby impacting water levels and biochemistry of vegetative communities more consistently within the ENP.

#### Freshwater wetlands.

Loxahatchee National Wildlife Refuge (LOX)- Alligators from LOX had the second largest niche among freshwater wetlands, only slightly smaller than WCA3 which was comprised of four sampling sites. The numerous tree islands and mosaic of habitats within LOX may provide alligators with additional sources of prey not available in the other freshwater sites, possibly resulting in a larger niche size due to the exploitation of additional food sources. Similar to WCA3, there are less restrictions in search areas resulting in greater temporal variability in capture locations and microhabitat’s biochemistry. Alternatively, LOX had the highest density of alligators among all sampled sites [92], therefore the larger niche may reflect an increase in diet breadth in response to increased competition. Furthermore, despite having the second-largest niche, alligators in LOX exhibited slightly lower body condition than those in WCA3, particularly among larger individuals. Thus, the increase in niche may instead be due to alligators expanding their foraging to less preferred areas or prey as a result of competition pressures.

Water Conservation Area 3 (WCA3)- The lower δ^15^N values detected in alligators sampled in WCA3 compared to those in ENP-FW were consistent with findings by Inglett et al. [89], which showed that total nitrogen levels of waters and soils were higher in Shark River Slough within ENP than WCA3. However, we do not believe that alligators occupy different trophic positions in these habitats. Instead, the elevated nitrogen levels in alligators from ENP-FW likely reflect variations in resources and prey due to the different baseline isotopic values at each site [30–32,89–91,93]. While alligators’ δ^15^N values were on average lower in WCA3 than ENP-FW, there was significant variation among years and sites within the water conservation areas. Several factors could contribute to the increases in δ^15^N values, including stress. Although some alligators exhibited slightly lower body condition values associated with higher nitrogen levels, most did not appear malnourished, suggesting that fasting stress was not the sole cause of the elevated levels. Research comparing Gambusia across several southern Everglades wetlands found significant spatial variability in both carbon and nitrogen isotopic ratios within each management area [94]. Consistent with our results, the study reported significantly higher δ^15^N values in the northwest and southwestern portions of WCA3B compared to the eastern. A possible explanation for the increased δ^15^N values in alligators may be related to prey higher δ^15^N values along the western areas of WCA3B due to their closer proximity to canals and associated nutrient inflows. Previous studies reported elevated nitrogen levels in flora and fauna closer to inflows, such as canals, with transition zones between the impacted sites and those more interior [90,95]. Thus, higher δ^15^N values in WCA3B in Wyears 2014 and 2015 may be attributed to capture locations being closer to the canal along the western edge of WCA3B than in WYs 2017 or 2019, or from the other sites within the Water Conservation Areas, WCA3 (HD, N41, TW). Additionally, higher δ^15^N values may result from bioaccumulation due to an increased diet of higher-trophic prey, such as carnivorous fish often associated with the deeper water canals that move between marsh and canals in response to fluctuations in water levels [96]. Future studies could include resource analysis as well as sampling alligators from canals to address these questions.

Big Cypress National Preserve (BICY)- Characterized by cypress swamps and freshwater marl prairies, BICY has diverse terrestrial and aquatic vegetation including periphyton, which plays important role as primary producer and as food for several of the alligator’s prey. Periphyton is ubiquitous with the Everglades marshes, with responding quickly to environmental changes resulting in a wide range of isotopic values [97]. Increases in periphyton biomass in response to hydrologic conditions can lead to increasing ^13^C, which can be further increased with high temperatures [98], which may help explain alligators’ elevated δ^13^C values. Unlike BICY, which is predominantly rain driven, WCA3 and ENP-FW receive canal water inputs that often contain elevated nutrient levels, including phosphorus. These elevated phosphorus levels can shift the composition away from periphyton communities, affecting community composition, periphyton biomass, and cover [97], which may lead to lower ^13^C levels. In the phosphorus-limited, oligotrophic Everglades, higher phosphorus often correlates with increased cattail strands, which are common in other freshwater wetlands but were not as dense near BICY capture sites. This suggests that BICY may have lower phosphorus levels and, consequently, may help explain the higher δ^13^C values compared to other freshwater wetlands. Additionally, alligators sampled from BICY were sampled within sawgrass sloughs surrounded by cypress forests, as well as near the mangrove ecotone on the western edge of the preserve. The alligators with the highest δ^13^C values were often caught closer to the coast potentially foraging on estuarine fishes or invertebrate prey with higher δ^13^C values within the mangroves, with those further inland having lower δ^13^C values (S6A Fig). Together these factors may have contributed to the higher δ^13^C values detected in BICY compared to the interior freshwater marshes within ENP-FW or WCA3. The isotopic niche of alligators was smallest within BICY, which also exhibited the most consistent niche geometry, suggesting stable resource use over the years. However, the similarity in niches may also be attributed to permit restrictions that limited search areas each year, in contrast to the unrestricted access in the four sites in the Water Conservation Area 3 wetland, which showed the most varied niches between years.

Everglades National Park freshwater marshes (ENP-FW)- Within the Park, δ^15^N values were generally higher in the same areas where δ^13^C values were lowest, extending southwest from ENP-FC to ENP-SS, while lower δ^15^N values were detected to the northwest and southeast (S6B Fig). This pattern was also observed in Gambusia and periphyton, where the study suggested that the elevated δ^15^N values resulted from denitrification in the sediments, leading to dissolved organic nitrogen with higher δ^15^N values that are incorporated into the biomass [94]. Of the three sites, ENP-NESSE was consistently drier and had higher δ^13^C and δ^15^N values than ENP-FC and ENP-SS, potentially contributed to by the drier conditions [99]. ENP-NESSE is characterized by exposed rocky marl calcium carbonate rich soils with little to no periphyton, muck layer, or marsh vegetation (Sidney Godfrey, University of Florida, personal observation), whereas ENP-FC and ENP-SS contain peat-dominated soils. ENP-NESSE experienced complete dry-downs during each dry season between Wyears 2017–2020. These dry downs can alter food webs [30] and the isotopic composition of plants [99], leading to reduced periphyton productivity and increasing rates of mineralization and nitrification, which affect both the carbon and nitrogen in the system [89]. As such, changes in δ^13^C in ENP-FC and ENP-SS may reflect vegetative shifts in microtopography and a reduction in community distinctness [100]. To help mitigate dry downs the Tamiami Trail bridge project is designed to increase water flow to NESSE and will help to keep the area flooded during the dry season. Since the initial one-mile section of the elevated Tamiami Bridge project came online in 2013 with another 2.3 miles of elevated bridge completed by 2019, we have observed a gradual increase in periphyton and a transition to basal sources more similar to ENP-FC. This likely has led to the increase in the number of alligators we have detected during seasonal monitoring surveys since the initial section was completed. We have not collected samples from ENP-NESSE since 2019, but we hypothesize that, with continued restoration and succession of aquatic vegetation, the niche of alligators sampled in ENP-NESSE may shift to resemble those from ENP-FC and ENP-SS due to more similar vegetative sources within ENP-NESSE. Like BICY, the higher consistency in alligator niches within ENP-FW sites may be attributed to their locations within a National Park, which restricts our sampling to alligators near the same trails and open sloughs each season.

#### Estuarine wetland.

Everglades National Park Estuary (ENP-EST)- The wider range of δ^13^C values from alligators within the estuary, compared to those in the freshwater wetlands, suggests that they are likely feeding from both freshwater and estuarine food webs. Estuarine primary producers and consumers tend to be more enriched in δ^13^C relative to freshwater primary producers [101], and the range of δ^13^C values in our study was consistent with those from nearby mangrove forests and estuarine resources − 27.3 ± 0.9 (− 29.2 to − 25.7), [15,36,102]. While alligators within the estuary had a wider range of δ^13^C values, it is important to note that a wide niche δ^13^C versus δ^15^N need not be associated with a broader diet as estimated by the number of different prey species utilized [103,104] and instead may indicate foraging across habitats or basal carbon sources. The enriched δ^13^C values (i.e., > 25.0–20‰) of larger alligators indicate they are foraging in more marine food webs, likely making trips downstream consuming larger predatory fish or other marine prey. This is in contrast to the medium-size alligators that appear to remain upstream near the headwaters to avoid increases in salt stress, likely foraging on smaller herbivorous fish or freshwater marsh prey with less enriched carbon and nitrogen values. Thus, estuarine alligators near the headwaters may access adjacent marshes foraging on freshwater food webs when salinities are too high, returning the estuary once salinity levels drop. This supports previous studies indicating that large alligators consume prey from both freshwater and marine sources, providing nutrient linkages between wetland ecosystems [15,41]. Additionally, alligators sampled within the estuary exhibited the largest overall niche and the most temporal variation, likely due to cross-ecosystem foraging influenced by tidal patterns and freshwater marsh [14,15,36,42]. During our study, estuarine alligators’ isotopic values were within similar ranges detected in previous studies [14,15,36], suggesting long-term stability in the use of different resource pools. While this indicates alligators generally maintain similar long-term foraging patterns, our results revealed significant shifts in niche size and position between seasons and Wyears, with fluctuations in both δ^13^C and δ^15^N values. These temporal changes are likely influenced by varying environmental factors that drive resource availability between seasons and years [14,19,42,105]. While Rosenblatt and Heithaus [15] used passive acoustic telemetry along with stable isotopes to identify specialized behavioral patterns in foraging and movements that resulted in consistent δ^13^C values, they only sampled adult males. Our study included females and sub-adult (medium) individuals, which may introduce size and sex related variables, as discussed in subsequent sections.

### Influence of intraspecific groups

#### Sex.

Our results indicated alligators’ carbon and nitrogen isotopic ratios and niches vary by sex, with the extent and direction of these differences and resulting niche overlap being influenced by capture site. Subsequent univariate analysis determined that differences were primarily driven by carbon, while both sexes exhibited similar nitrogen values. This suggests that the univariate analysis for δ^15^N alone may not capture these effects due to correlations with δ^13^C or the specific nature of the isotopic responses. Consequently, males have wider niches characterized by less overlap in carbon, and more similarity in nitrogen values. The differing carbon values imply that males, which typically have larger home ranges and utilize additional habitats [23,42], leading to reduced carbon overlap with females.

Similar to a 2022 study within WCA3B [106], mean isotopic values of males in our study were slightly higher in both δ^13^C (−27.04‰) and δ^15^N (8.81‰) values than females (−27.37‰, 8.75‰ respectively) but statistically similar. Unlike the previous study, we also compared the isotopic niches between sexes within each of the wetlands/sites and found that male niches often differed in geometry, position in δ-space and had a larger niche (SEA_B_: F < M = 0.6065). This demonstrates the value of comparing isotopic niches as it can reveal patterns in the spread of values representing the breadth of resources utilized within a population that may not be detected when comparing mean values. Our results agree with previous alligator studies which detected varying degrees of niche overlap between sexes in salt marshes of northeastern Florida and estuarine impoundments in coastal Georgia, which were dependent on sampling site and Wyear [19,41]. Males occupied a larger niche than females, particularly within the estuary, with only those from WCA3 having similar sized niches. The wider range in δ^13^C versus δ^15^N values suggest utilization of additional habitats or basal sources are driving the larger and more varied isotopic niche of the males than females, which agrees with studies that demonstrated male alligators have larger home ranges year-round and overall greater movement rates than females [20,22,23,106]. Thus, as males likely fed on similar prey as females as they traveled through additional habitats, and those resources had different baselines or basal sources resulting in a wider range of δ^13^C values.

Reproduction can influence the isotopic values of female alligators both physiologically and behaviorally. In reptiles, physiological variations due to egg production and maternal transfer have been demonstrated in several species of sea turtles, where offspring are often enriched in δ^15^N and depleted in δ^13^C relative to their mothers [63,107]. Female alligator behavior during nesting changes as they reduce their movements to guard their nests until hatching, and will remain close for some time, potentially reducing their niche while caring for their young. Alternatively, shifts in male alligator niches may reflect isotopic labeling from other local food webs as they search for new habitat or mates. This suggests that a combination of physiological and behavioral differences between the sexes contributes to the niche characteristics we observed. Future studies incorporating resource sampling could clarify the proportion of resources used by each sex, helping to elucidate the extent to which physiological versus behavioral differences drive these variations. Additionally, we observed seasonal variations in resource use, with both sexes exhibiting larger niches with less overlap during spring captures (S4A Fig). These captures reflect alligator diets from the last months of the wet season to the beginning of dry season, coinciding with higher water levels. These conditions may allow for increased movements between habitats or enhance prey availability, potentially broadening their diet and isotopic niche. This is in contrast to the fall captures, where we typically observed increased niche overlap between sexes, driven by a decrease in the overall niche size of female alligators. This change correlates with lower water levels, which may limit movements and habitat availability, as well as it coincides with the onset of the breeding season [22,23,108,109] when both sexes forage near each other on similar resources. Future studies including resource sampling could clarify the species and proportions of resources used by each sex, thereby enhancing our understanding of the physiological and behavioral variations driving these differences.

#### Size class.

In this study, the mean bivariate response of δ^13^C and δ^15^N values was influenced by size class, which varied based on site, sex, capture period, and Wyear. Unlike the differences driven by carbon between sexes, univariate analysis revealed that nitrogen was the primary factor driving the observed differences between size classes, with large alligators, on average, having higher δ^15^N values than medium-sized alligators. From a biological perspective, size class may significantly influence δ¹⁵N due to dietary differences while foraging in similar habitats, suggesting that δ¹³C may be less sensitive to size or influenced by other variables. This indicates that within a given site, capture season, and water year, both size classes may forage from similar food webs; however, larger alligators may target higher trophic prey or consume a different proportion of dietary resources, leading to higher nitrogen enrichment, while medium-sized alligators may consume more herbivorous aquatic prey with lower δ^15^N values.

Similar to previous studies on crocodilians that have detected ontogenetic shifts between size classes [19,38,41,110], our results also suggested ontogenetic shifts for those in estuarine environments. Although the findings for freshwater wetlands were less direct, hatchlings and juveniles were not included in our samples. Therefore, it is possible that by the time alligators within the marsh reach 1.5 meters, their diets may be similar, differing only in proportions, or supplemented by additional sources.

The isotopic niches indicate that both size classes of alligators have similar carbon values, further suggesting foraging in comparable locations, while nitrogen values show less overlap. This pattern implies resource partitioning, with larger alligators occupying higher positions in the niche and foraging on a greater proportion of nitrogen-enriched prey from higher trophic levels, particularly in estuarine environments where salinity limits resources for medium-sized alligators. Larger individuals also occupied a slightly larger niche with a wider range of carbon values and enriched nitrogen isotopic values compared to medium sized sub-adult alligators, although both size classes were within the same tropic level.

The smaller niche of medium alligators suggests more specialization, potentially arising from competitive pressures from larger alligators that restrict them to smaller resource pools. While we likely did not have a large enough sample size to detect significant differences in the niches between size classes, greater niche overlap often corresponded with a higher body condition for medium-sized alligators and lower body condition for larger alligators. This may be due differing energetic needs required to maintain body condition. When niches overlap, it could indicate limited resources during seasonal drying events, allowing medium-sized alligators to maintain a higher body condition compared to larger alligators.

### Temporal variations

The results of the MANOVA, particularly the interaction term (Season * Site * Wyear), indicate that stable isotope ratios differ among sites, influenced by capture season and water year. Seasonal fluctuations in precipitation, temperature, light, and nutrients drive resource availability across Everglades habitats [27,30–32]. Within each site, seasonal variations in the isotopic composition of dissolved inorganic carbon and nitrogen in the water column can affect the δ^13^C and δ^15^N values of plants at the base of the food web [91,111]. While turnover rates in alligator whole blood may represent slightly longer time scales than one wet/dry season, differences in isotopic values and niches were detectable between capture seasons. This suggests there was enough of a shift in core use to be detectable, making these seasonal comparisons more informative for our models than yearly intervals. Additionally, although plasma and whole blood have different turnover rates potentially influencing their niche characteristics, we observed similar seasonal and annual trends regardless of tissue type. This congruence supports the idea that seasonal variations in alligators’ diets persist for several months, allowing sufficient time between captures for isotopic incorporation to detect seasonal shifts in both tissues. Whereas the longer turnover rates from alligator tissues prevent us from relating values or niches back to a specific time window, the differences detected likely reflect influences from seasonal dietary variations. Thus, spring captures reflect foraging during the end of the wet season into the beginning of the dry season, while fall captures reflect foraging during the end of the dry season into the beginning of the wet season, which includes the breeding season. Annual wet/dry season fluctuations typical of these wetlands may require animals (both predators and prey) to move between habitats, potentially leading to the food-web changes we documented. The timing and duration of each wet/dry season cycle various annually (S7 Fig), which can influence the length of time for the water levels to recede impacting alligator behavior, diet, and body condition [27]. Platt et al. [112] found that while adult and juvenile alligator diets did not differ significantly in prey specie, they exhibited seasonal differences possibly due to fluctuating water levels; similar seasonal variations in resource us, isotopic values, and isotopic niches have also been detected in alligators within the Everglades in both estuarine [14,42] and freshwater wetlands [13,106]. In the estuary, dry-season salinity levels can lead to behavioral changes, with alligators spending less time downstream due to physiological stress or because the potential foraging benefits do not outweigh the costs [15]. However, larger alligators likely still utilize downstream areas despite this stress to access greater prey resources, as indicated by their relatively higher δ^13^C and δ^15^N values compared to medium alligators.

Existing within in a seasonally pulsed wetland between dry and wet seasons, alligators of both sexes within our study sites exhibited niche shifts between seasons. Yet, none of the shifts appeared to result in higher trophic position represented by an increase in stable nitrogen isotope ratios, and instead reflected a shift in stable carbon isotope ratios (Fig 4). This agrees with the spatiotemporal shifts in diets and trophic niche exhibited by fishes within the Everglades, where their results indicated omnivory seems variable in its direction and magnitude and more likely to result in a change in diet and niche breadth than trophic position [32]. Alligators sampled during the spring captures often occupied larger niches than those captured in the fall. Spring captures reflect diets from several months prior during the wet season, when higher water levels likely allowed for increased movements and resource availability (S7 Fig). Additionally, these captures occurred before nesting, meaning females had not yet restricted their movements or foraging due to nest guarding. Our results support this, showing that mean differences in niche size between capture seasons were driven by females’ smaller niches in the fall than spring captures, while male niches were slightly larger during the fall captures but statistically similar (S4A Fig and S8 Fig). This contrasts with fall captures which reflects diets from the previous dry season, when lower water levels likely restricted movements and prey availability. Furthermore, fall captures occur after the breeding season, when both sexes likely foraged in similar habitats on similar resources, and females caring for their young may have further limited their movements and resource availability resulting in smaller niches than males (S8A and S8B Fig). As previously stated, the highest level of consistency in shape and position (and thus resource use), as well as overall estimated niche size (SEA_B_) over time, was observed in sites with restricted search areas (BICY and ENP). conversely, sites with larger, unrestricted search areas (EST, LOX, and WCA3) exhibited greater variability among years due to sampling in more diverse microhabitats within these areas (S9A–S9E Fig). Although we were unable to sample enough size classes from all sites, a general pattern emerged. There was greater overlap between size classes during the fall sampling period, which reflects foraging when water levels were lower. This likely reduced prey diversity and increased the likelihood of both size classes foraging within similar areas. Whereas the spring sampling period following the wet season, when higher water levels may have led to increased prey diversity and additional habitats, reducing competition for medium-sized alligators from larger alligators (S4B Fig). As such, the drier conditions at WCA3-TW compared to the other sites could be driving the overlap in size class niches during the dry season because they are forced into the same locations or to use the same resources. That is, they have less areas and thus variety of resources to use during the dry season at WCA3-TW than they might in the other sites. Alternatively, the more drastic drying conditions may be limiting the resources making their niches more similar between seasons than at other sites. Given the longer turnover rates for tissues analyzed and small sample size of medium and small animals in this study, additional investigations into the drivers of seasonal variations could benefit from utilizing tissue with shorter turnover rates (plasma) as well as including resource sampling within each site and season across multiple years.

### Influence of intraspecific niche characteristics on body condition

Alligators in sites with more consistent niche geometries (BICY, ENP-FC, and ENP-SS) exhibited more stable body conditions across water years compared to sites with greater temporal variation, such as those within WCA3 and ENP-EST. This finding suggests that when resource abundance and availability are relatively stable, alligators’ dietary breadth‒ described by their isotopic niche (δ-space)– and overall body condition tends to remain consistent. For example, alligators sampled from the four sites in the WCA3 wetland experienced the most significant shifts in niche geometry as well as mean body condition values between years, likely due to fluctuating resource availability or movements between habitats attributed to the variability in hydrologic and nutrient conditions within.

An alligator’s sex and size class can affect stable isotope (SI) ratios and body condition (BC), though the underlying processes may differ. Sex and maturity affect both SI ratios and body condition through differences in energy allocation (growth or reproduction), metabolic changes, foraging behavior, physiological stress, social dynamics, and health factors [19,53,56,57,64]. Behavioral differences in home ranges and movement patterns between sexes and size classes can also lead to foraging in different habitats or from different food webs influencing isotopic ratios and potentially available resources [21,23,42]. In this study, alligator body condition values for both sexes were often similar within a given wetland or site during each Wyear and capture period; however, their niche geometry and percent overlap often varied, with males generally being larger (S3 Table, S4A Fig). These differences suggests that males and females utilize different proportions of resources or habitats to achieve similar body conditions. We found that mean δ^13^C or δ^15^N values, as well as overall niche size within a site, were not directly correlated with body condition, likely due to differences in habitats, basal sources, and isotopic ratios at each site. Instead, our models suggest that changes in the dietary breadth resource use similarity between sexes during the 4–6 months prior to capture can influence whether body condition increases or decreases.

As niche breadth (i.e., resource utilization) increases, body condition improves, particularly when male and female niches have greater overlap while maintaining dissimilar core niches, as indicated by greater centroid distances. These increases suggest sufficient resources are available for both sexes to increase their fat reserves, indicating a healthy ecosystem [52]. Conversely, when the centroids of their niches are closer together, with smaller niches and less overlap, it may reflect an increased specialization or reduction in resource availability or abundance [68,69]. In these cases, both sexes may forage from smaller resource pools, likely including less preferred food sources, which could lead to decreases in body condition. When both sexes utilize similar resource pools, indicated by closer centroids but with smaller breadth and less similarity, body conditions decline. This may suggest that both sexes are foraging within same locations but with less diversity and possibly less energetically favorable prey, potentially due to seasonal fluctuations in water levels and prey availability. Although the relationship between body condition and breeding is not fully understood, preliminary information suggests that nesting females have higher body condition at the beginning of nesting than non-nesting alligators [27]. Additionally, males’ niche sizes remained relatively stable between seasons, while females exhibited larger niches in the spring capture period and smaller niches in the fall following the nesting period. These observed differences in female niche size may be attributed to physiological (egg formation) or behavioral (nest guarding and reduced movements) factors not exhibited by the males. For example, female δ¹⁵N values may increase due to fasting during nest guarding, which can also lead to decreases in body condition [19].

Differences in metabolic rate between size classes can influence both stable isotope (SI) ratios and body condition (BC) through differences how efficiently these individuals convert food into body mass. Smaller individuals typically exhibit faster isotopic turnover than larger, older individuals, thus incorporating SI values more rapidly [56,57,60]. Although not statistically significant, large alligators had larger niches than medium-sized alligators, suggesting they utilize a broader diversity of resources or habitats. Additionally, large alligators often exhibited better body condition, which increases with niche overlap. Larger alligators can access a wider diversity of prey, potentially expanding their niche, while competition may force medium alligators into smaller niches or less optimal foraging. This affects their SI ratios, as larger alligators have broader ranges of carbon and nitrogen values, while the increased competition or foraging on lower trophic (lower δ¹⁵N) or nutrient-poor prey may lead to lower body condition in medium alligators. However, this only remained true in sites with relatively low densities of alligators. For example, body condition was frequently higher for large alligators than medium alligators in ENP-FW, WCA3, and EST which all had relatively lower densities with alligators often caught alone [92]. This contrasts with those in LOX, where alligator densities were much higher, and medium sized alligators exhibited higher mean body condition values than larger ones regardless of capture season (S3 Table). The elevated δ^15^N values of larger individuals within LOX, relative the medium-sized alligators, may indicate increased stress, supporting this possibility. Future research could further investigate whether large-sized alligators experience similar competitive pressures against each other at high densities, as medium-sized alligators do against large alligators at lower densities.

Despite the Everglades’ year-round temperatures favorable for alligators’ growth [113], limited food availability [30,32,93] can counteract these benefits [25]. With decreased productivity, energetically favorable resources can become scarce. Therefore, to meet metabolic demands, species must expand the breadth of their niche to include additional habitats and/or less favorable resources. Larger niches resulting from generalist foraging strategies that incorporate these additional habitats, or less favorable resources can increase a species’ fitness [66,67]. For example, a previous study suggested that alligators likely used downstream areas within ENP-EST, despite salt stress, to access greater prey resources during the wet season when salinities are lower [15]. Conversely, when niches contract, it may reflect a reduction in prey availability, leading to relatively more specialization than when the niche was larger, resulting in decreased body condition as choices become limited. As overlap decreases, body condition continues to decline, suggesting that the sexes are utilizing different proportions of available prey or altering their movements to find resources, thereby incorporating values from other biochemical baselines. For instance, in Wyear 2014, there was the largest percentage of alligators in poor condition, associated with a wider range and higher δ^13^C values following drought conditions in south Florida [100]. However, 2019 was an outlier, with the highest percentage of alligators in excellent condition and the lowest in poor condition, coinciding with relatively smaller niches in most wetlands and sites, which may warrant additional research as sample size was smaller during that year. Although the absence of resource data limited our ability to identify the drivers of the observed changes, fluctuations in niches and body conditions across years suggest intraspecific seasonal shifts among all sites is important to note that dietary shifts indicated by stable isotope ratios may not directly correlate with changes in body condition, as other factors, such as habitat quality and competition, also play significant roles. Therefore, future investigations that incorporate resource sampling could help elucidate the factors driving the variations we observed.

## Conclusion

Our findings demonstrate the spatiotemporal plasticity of American alligators’ isotopic niches across the southern Everglades, with different demographics occupying distinct niches that exhibit varying overlaps in diet. Analyzing data at a smaller scale (sites) provided more informative models than at larger scales (wetlands), reflecting the habitat compartmentalization, nutrient availability, and hydrologic differences within each site. Despite similar resource use and body conditions across sexes and size classes, significant variations in alligators’ isotopic niches suggest differing prey utilization influenced by fluctuations in hydrology. Future studies could also benefit from incorporating seasonal hydrology data and resource availability assessments. Although diet reconstruction was not the focus of this study, subsequent isotopic studies that incorporate local resource samples from six months to a year prior, along with alligator plasma samples, could help clarify the contributions of prey species to the observed niche dynamics and, consequently, body condition. Given the variability in intrapersonal niches, landscape heterogeneity and dynamic hydrology are considerations for sustainable management and conservation efforts aimed at maximizing alligator foraging and overall fitness.

## Supporting information

S1 Appendix Correlations among Everglades alligator populations’ δ^13^C and δ^15^N values. (DOCX)

S1 Fig Tissue adjustments and estimated timeline. Original versus adjusted isotopic niches calculated from 38 paired plasma and whole blood samples collected from alligators in 2016. A) Isotopic niches represent original raw data, B) Isotopic niches after applying correction factor to plasma samples. C) Estimated timeline for body condition and isotopic assimilation in relation to body condition. Hatched areas reflect earliest assimilation dependent upon initial captures during each capture period.
(TIF)

S2 Fig Boxplots of alligator body condition and corresponding isotopic values. Range of isotopic values and corresponding alligator body condition for each water year byA) δ^13^C and wetlandB) δ^13^C and site, C) δ^15^N and wetland, D) δ^15^N and site.
(TIF)

S3 Fig Alligators isotopic niche comparisons among the multiple sites within the ENP-FW and WCA3 wetlands. American alligator (*Alligator mississippiensis*) estimated 40% core isotopic niches from A) five sampled wetlands of the southern Everglades ecosystem, Florida USA. Mean values are shown by solid dot, with each ellipse illustrating the 40% core isotopic niche within each wetland. B) Bayesian estimated standard ellipse area (SEA_B_) niche size for each wetland. C) American alligator (*Alligator mississippiensis*) isotopic means and estimated 40% core isotopic niches from each of the sampling sites within the ENP-FW and WCA3 wetlands illustrating the variability within each. D) SEA_B_ size for each sampling site within the ENP-FW and WCA3 wetlands. Boxes represent 50%, 75%, and 95% credibility intervals, and black dots correspond to the median.
(TIF)

S4 Fig American alligator (*Alligator mississippiensis*) seasonal intraspecific isotopic niche. Estimated 40% core isotopic niches for each site and capture period (season) by A) Sex; blue triangles and ellipses represent male alligators and red circles and ellipses represent females, B) Size class; green triangles and ellipses represent medium alligators (Total Length (TL): 1.25> <1.75m), red circles and ellipses represent large alligators (TL: ≥ 1.75 cm).
(TIF)

S5 Fig American alligator (*Alligator mississippiensis*) boxplots depicting body condition by sex. Range of Fulton’s K values among A) Wetlands and water years, B) Sites and water years.
(TIF)

S6 Fig Alligator captures by isotopic values (all years). Capture locations of American alligator (*Alligator mississippiensis*) within the southern Everglades ecosystem, Florida, USA depicted by isotopic values of A) δ^13^C and B) δ^15^N during this study.
(TIF)

S7 Fig Mean water levels recorded by gauges within each study site. Values are reflective of the water surface elevation relative to a reference point, known as “stage,” expressed in feet. Data obtained from Everglade Depth Estimation Network (EDEN) for Support of Biological and Ecological Assessments. (Stage ID: LOX7_1587589081, TW-andy_1587589579, HD-site64_1587589966, N41site65_1587590071, 3B_1587589656, SS-NP203_1587590145, FC-NESR1_1587590219, NESSE_1587597146, BICY_1587504468, SR-gun_1587589447. https://sofia.usgs.gov/eden/stationlist.php).
(TIF)

S8 Fig American alligator (*Alligator mississippiensis*) isotopic niche between capture periods. Estimated 40% core isotopic niches for each capture period (season) by A) Sex; blue circles and ellipses represent male alligators and red circles and ellipses represent females, B) Bayesian estimated niche area SEA_B_ for each sex and capture period. Boxes represent 50%, 75%, and 95% credibility intervals, and black dots correspond to the median.
(TIF)

S9 Fig Alligator captures by isotopic values during each water year. Capture locations of American alligator (*Alligator mississippiensis*) within the southern Everglades ecosystem, Florida, USA depicted by isotopic mean δ^13^C (left) and δ^15^N (right) values by water year A) 2014, B) 2015, C) 2016, D) 2017, E) 2019 *Note, not all sites were sampled each water year.
(TIF)

S10 Fig Isotopic correlation plots by sex, size and site. Relationships of (A) δ^13^C and (B) δ^15^N with the sex-SVL interaction from 688 capture event tissue samples from 647 American alligators within the southern Everglades ecosystem, Florida, USA during this study (*Excludes 5 individuals where sex was not determined). The ten sampling sites were within five wetland watersheds, Arthur R. Marshall Loxahatchee National Wildlife Refuge (LOX), Big Cypress National Preserve (BICY), estuaries within southwestern Everglades National Park (ENP-EST), and multiple sites within Water Conservation Area 3 (WCA3; WCA3A-TW (Tower), WCA3A-HD (Holiday Park), WCA3A-N41, WCA3B-3B), and Everglades National Park freshwater marshes (ENP-FW; ENP-FC (Frog City), ENP-SS (Shark Slough), ENP-NESSE (NE Shark Slough).
(TIF)

S1 Table Contrasts cross-summarized of American alligator (*Alligator mississippiensis*) mean δ^13^C and δ^15^N within the southern Everglades ecosystem during water years 2014–2019 by Sex and Site. *Significant differences between sites are represented by dissimilar subscripts.
(TIF)

S2 Table Contrasts cross-summarized of American alligator (*Alligator mississippiensis*) mean δ^13^C and δ^15^N within the southern Everglades ecosystem during water years 2014–2019 by site, water year, and capture period (Spring or Fall). *Significant differences between sites are represented by dissimilar subscripts.
(TIF)

S3 Table Alligators’ Fulton’ k score and body condition by site and season 2012–2019. Fulton’s K equation used to generate body condition values [K = M/SVL^3^ × 10^5^], classified as Poor (≤1.95), Fair (>1.95– ≤ 2.1), Good (>2.1– ≤ 2.27), or Excellent (>2.27). Body condition values in **bold** were higher in spring capture period, those in *italic* were higher in fall capture periods, normal font no difference between capture periods. *Excludes alligators in the small size class and those whose sex was undetermined.
(TIF)
