# Supplementary material for: Isotopic niche plasticity of American alligators within the southern Everglades
Source: PLoS One. 2025 Jun 27;20(6):e0326148. doi: 10.1371/journal.pone.0326148 (PMC12204550; doi:10.1371/journal.pone.0326148)
Supplement: S1 Appendix — (DOCX) [file pone.0326148.s001.docx]

In the freshwater marshes, δ^13^C was positively correlated with size for male alligators in WCA3A-N41 and negatively correlated for females within WCA3A-TW; otherwise, no correlation was detected. For δ^15^N, males were positively correlated with size within ENP-SS and WCA3B-3B, while females showed positive correlations in ENP-SS and WCA3A-HD; otherwise, no correlation was observed (S3 Figs A and B). Males in ENP-EST had higher δ^15^N values than females, and while there were no significant differences in δ^13^C and δ^15^N values between sexes in the freshwater areas, male alligators’ δ^13^C and δ^15^N values in the estuary increased with size, while females showed no correlation. Isotopic values varied among all sampled sites (S10 Figs A and B) and varied dependent on Wyear (S9 Figs A-E) and capture period (S2 Table). Alligators’ δ^15^N values were often highest in ENP-FW, while alligators in LOX or WCA3 had the lowest values, depending on capture period and Wyear (S2 Table).
