# Supplementary figures and images for: Isotopic niche plasticity of American alligators within the southern Everglades
Source: PLoS One. 2025 Jun 27;20(6):e0326148. doi: 10.1371/journal.pone.0326148 (PMC12204550; doi:10.1371/journal.pone.0326148)

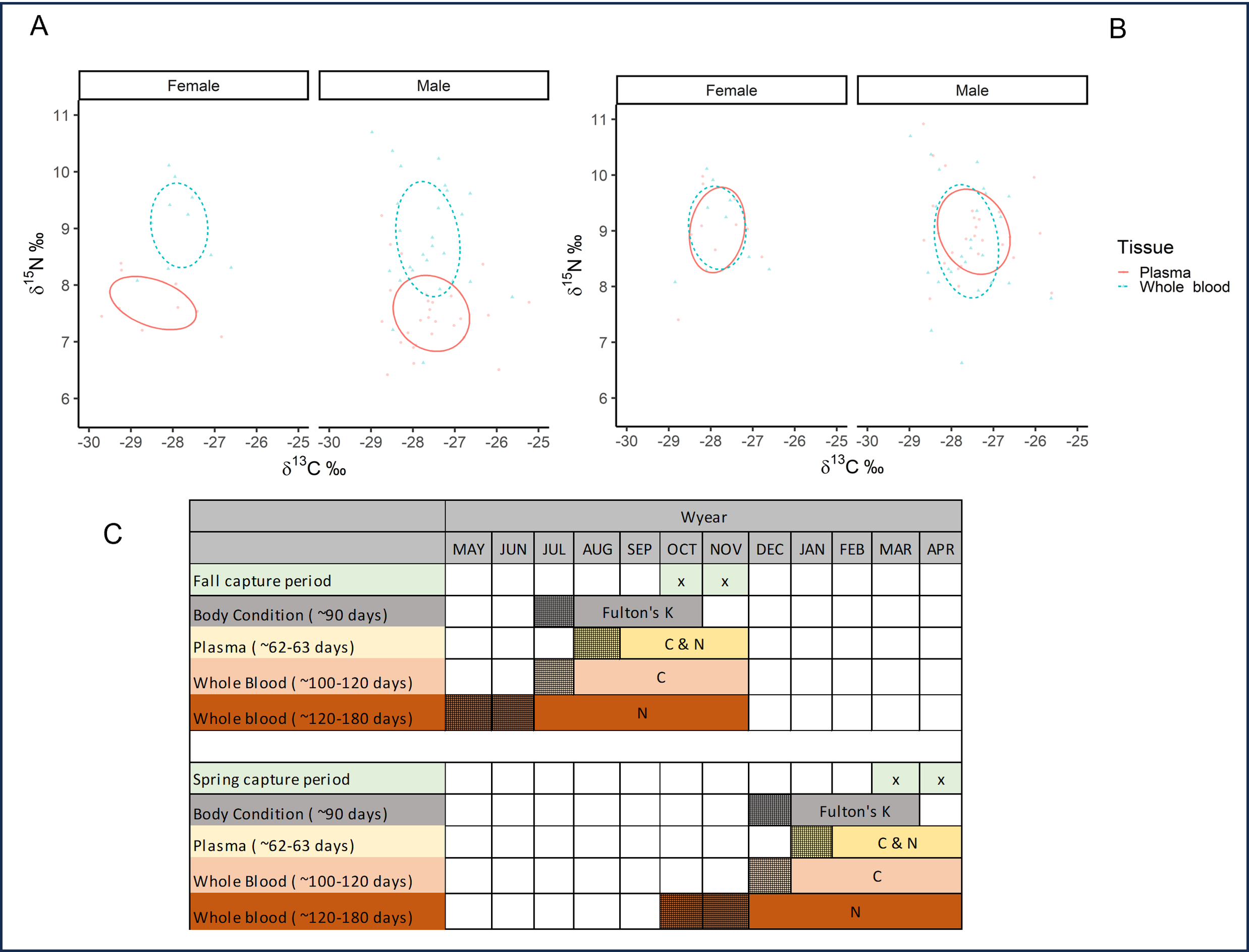

Supplement: S1 Fig — Original versus adjusted isotopic niches calculated from 38 paired plasma and whole blood samples collected from alligators in 2016. A) Isotopic niches represent original raw data, B) Isotopic niches after applying correction factor to plasma samples. C) Estimated timeline for body condition and isotopic assimilation in relation to body condition. Hatched areas reflect earliest assimilation dependent upon initial captures during each capture period. (TIF) [file pone.0326148.s002.tif]

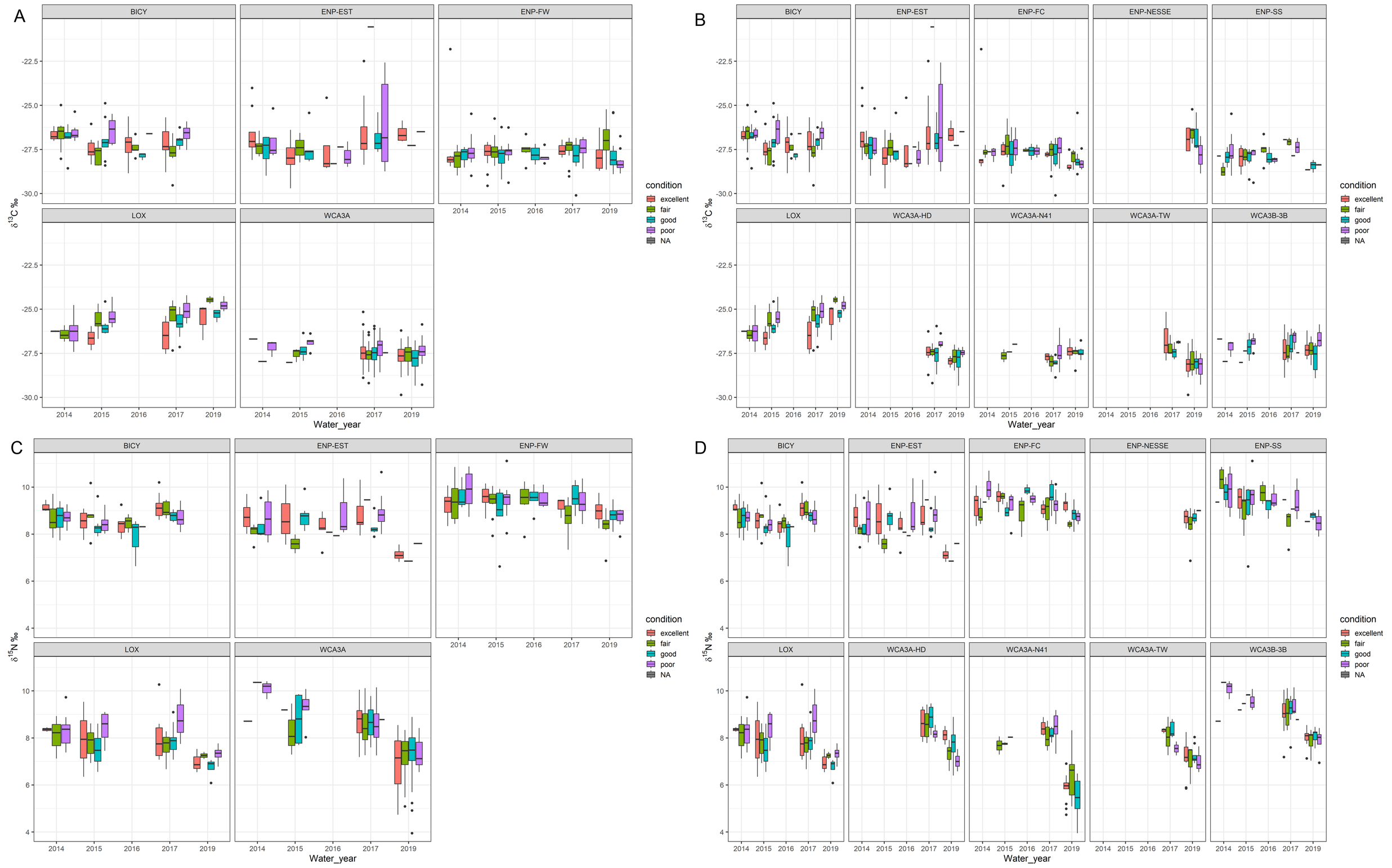

Supplement: S2 Fig — Range of isotopic values and corresponding alligator body condition for each water year byA) δ13C and wetlandB) δ13C and site, C) δ15N and wetland, D) δ15N and site. (TIF) [file pone.0326148.s003.tif]

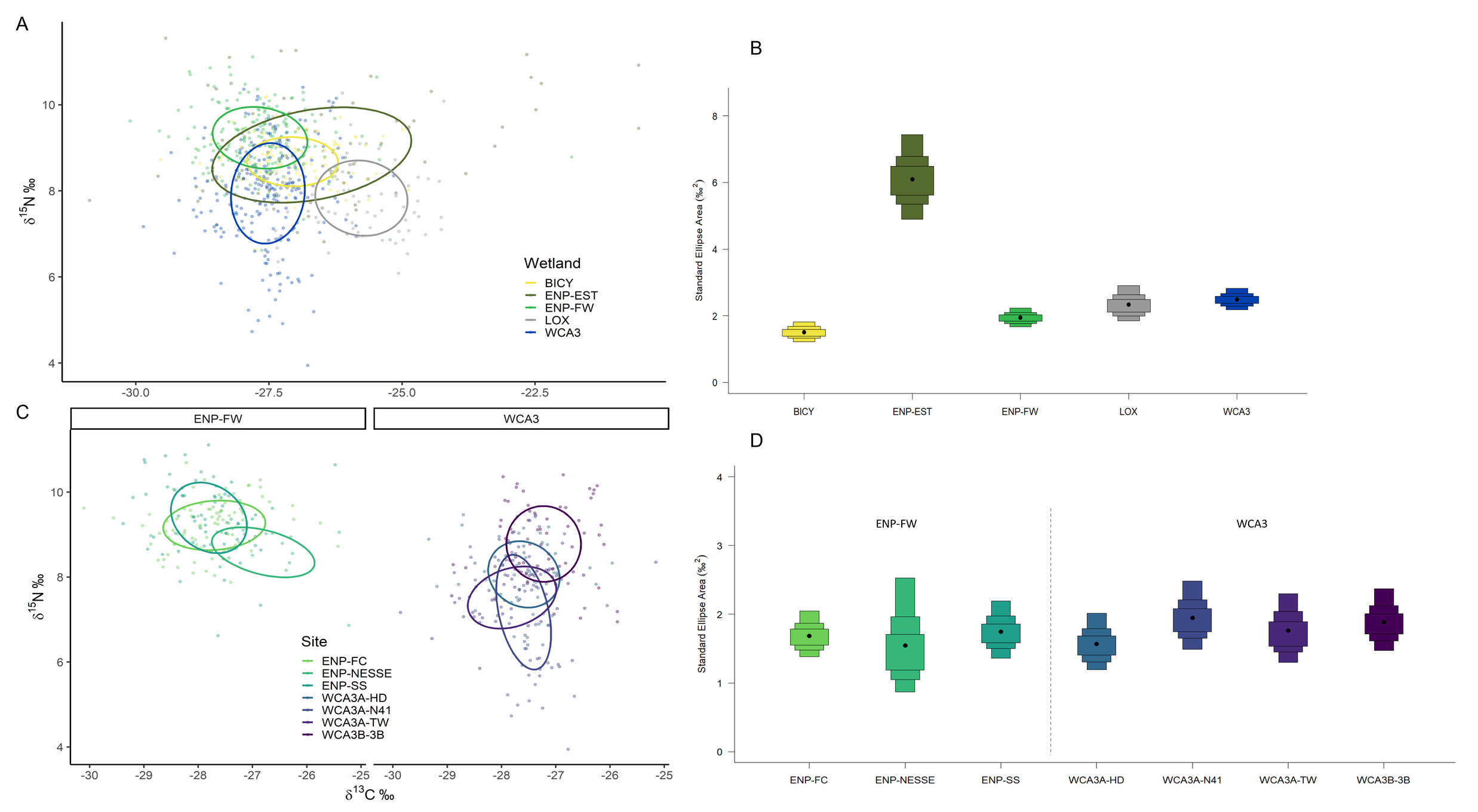

Supplement: S3 Fig — American alligator (Alligator mississippiensis) estimated 40% core isotopic niches from A) five sampled wetlands of the southern Everglades ecosystem, Florida USA. Mean values are shown by solid dot, with each ellipse illustrating the 40% core isotopic niche within each wetland. B) Bayesian estimated standard ellipse area (SEAB) niche size for each wetland. C) American alligator (Alligator mississippiensis) isotopic means and estimated 40% core isotopic niches from each of the sampling sites within the ENP-FW and WCA3 wetlands illustrating the variability within each. D) SEAB size for each sampling site within the ENP-FW and WCA3 wetlands. Boxes represent 50%, 75%, and 95% credibility intervals, and black dots correspond to the median. (TIF) [file pone.0326148.s004.tif]

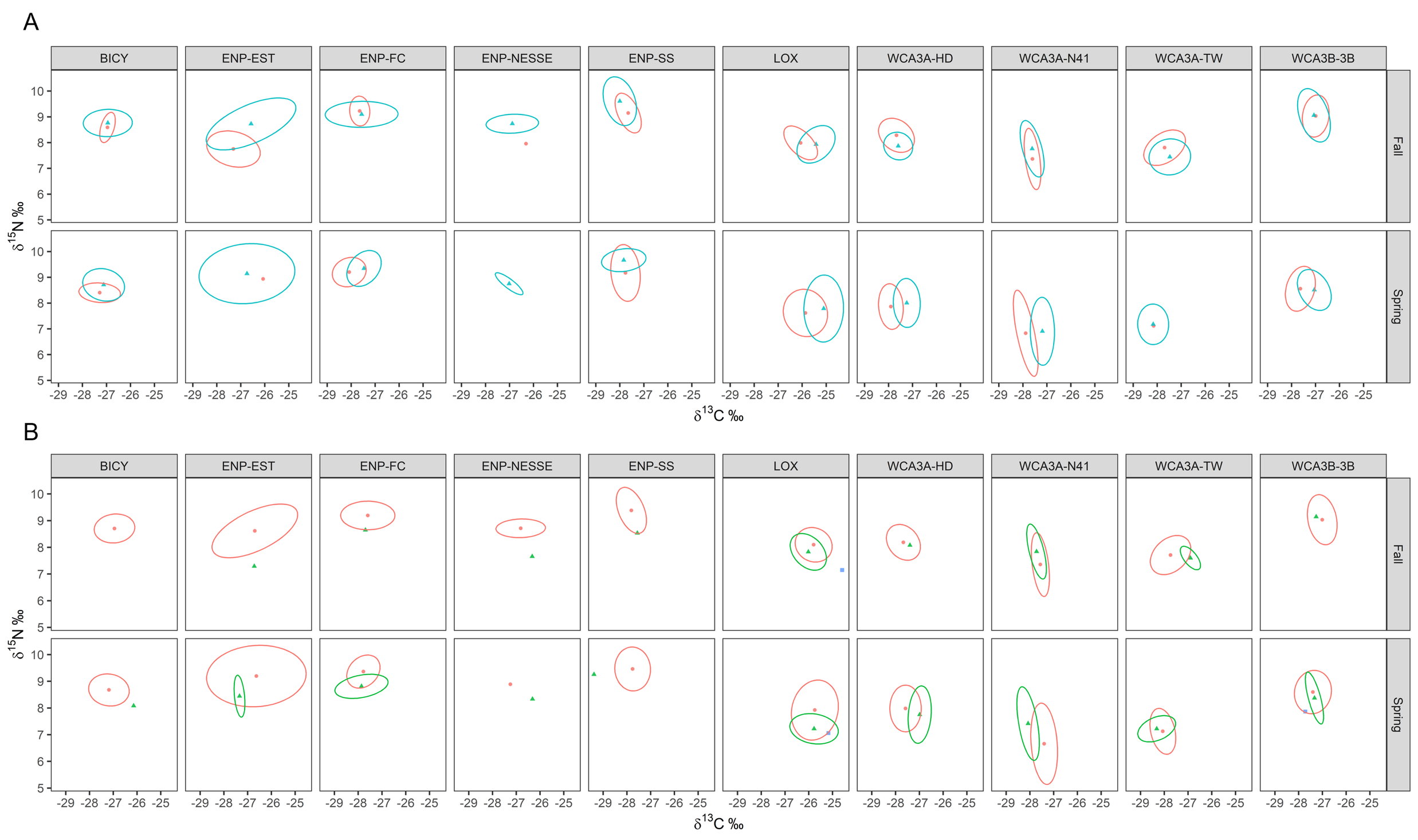

Supplement: S4 Fig — Estimated 40% core isotopic niches for each site and capture period (season) by A) Sex; blue triangles and ellipses represent male alligators and red circles and ellipses represent females, B) Size class; green triangles and ellipses represent medium alligators (Total Length (TL): 1.25> <1.75m), red circles and ellipses represent large alligators (TL: ≥ 1.75 cm). (TIF) [file pone.0326148.s005.tif]

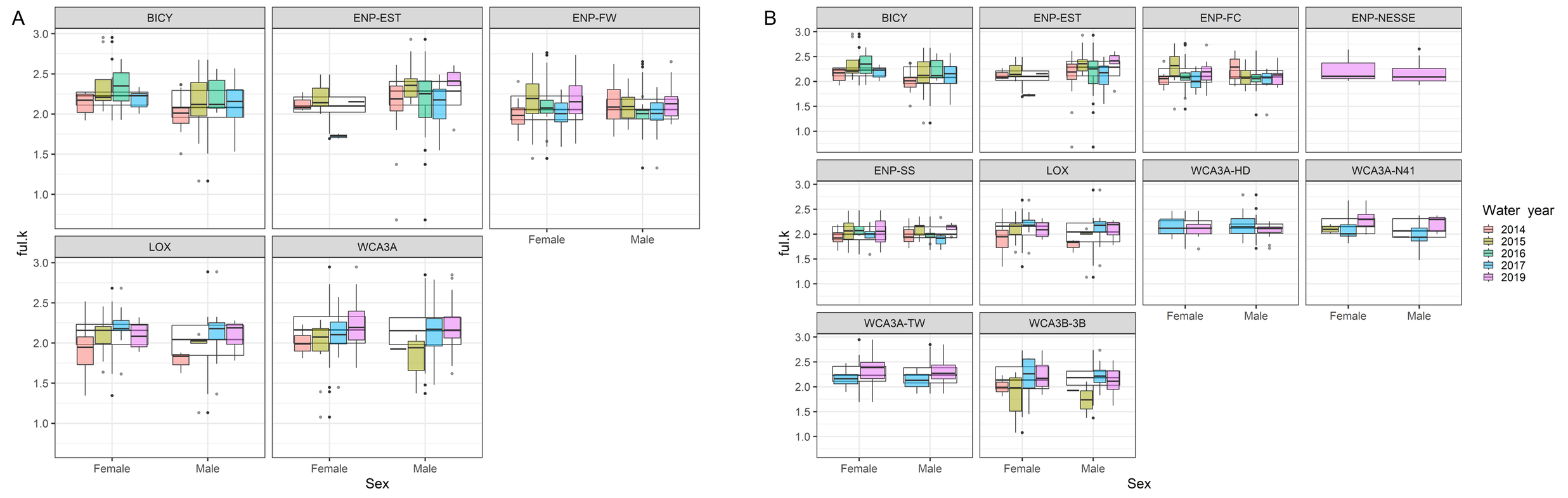

Supplement: S5 Fig — Range of Fulton’s K values among A) Wetlands and water years, B) Sites and water years. (TIF) [file pone.0326148.s006.tif]

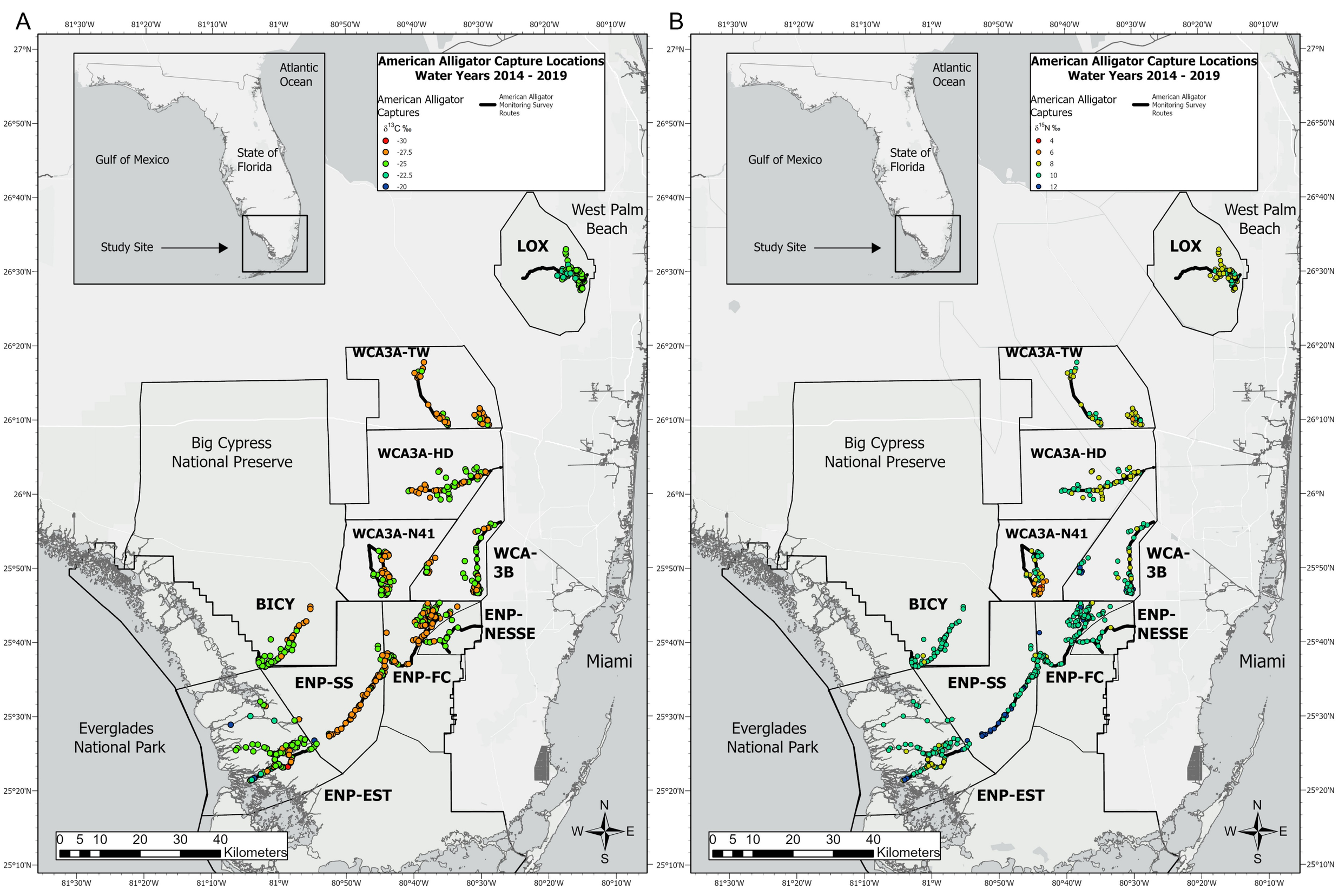

Supplement: S6 Fig — Capture locations of American alligator (Alligator mississippiensis) within the southern Everglades ecosystem, Florida, USA depicted by isotopic values of A) δ13C and B) δ15N during this study. (TIF) [file pone.0326148.s007.tif]

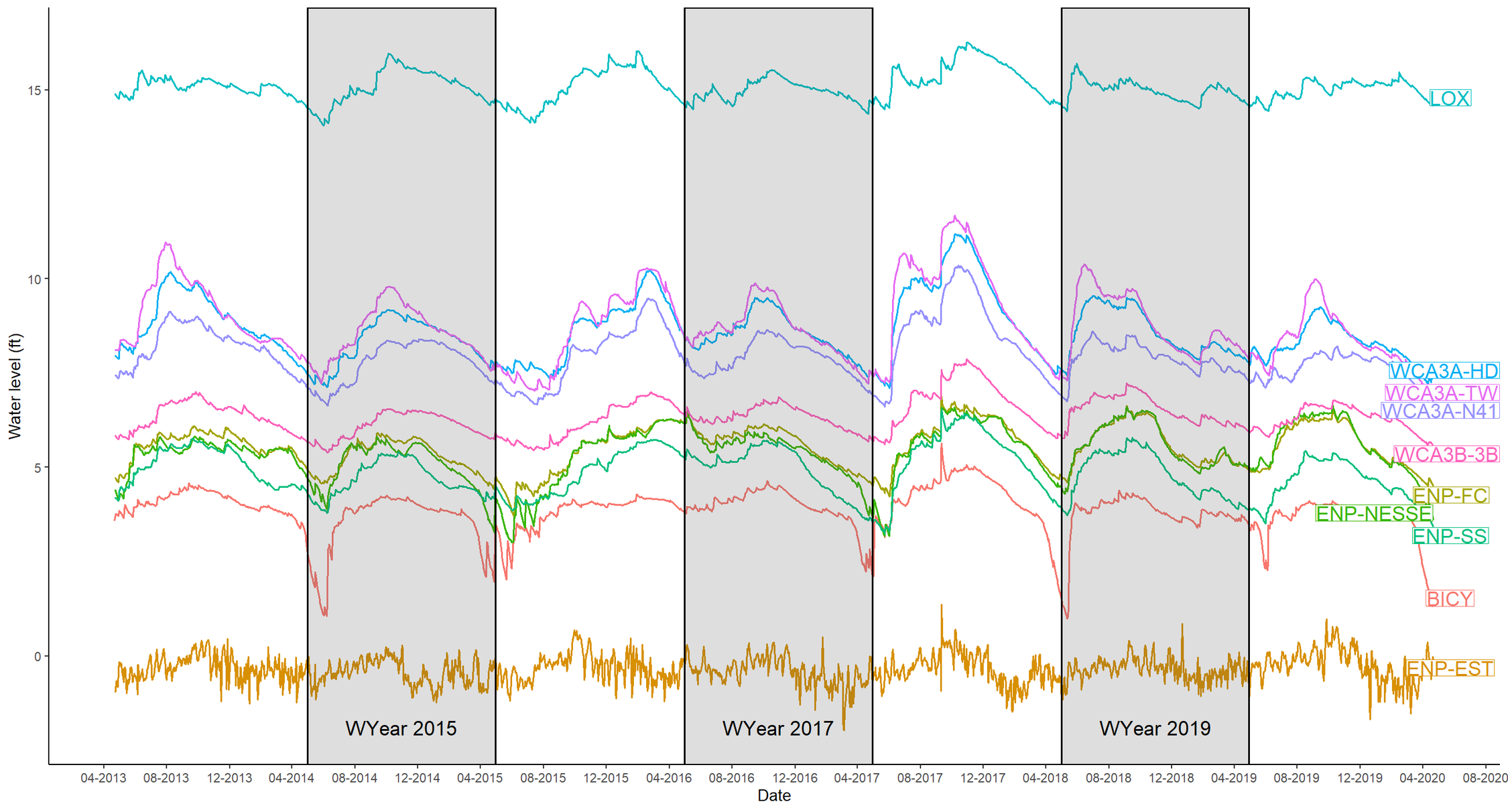

Supplement: S7 Fig — Values are reflective of the water surface elevation relative to a reference point, known as “stage,” expressed in feet. Data obtained from Everglade Depth Estimation Network (EDEN) for Support of Biological and Ecological Assessments. (Stage ID: LOX7_1587589081, TW-andy_1587589579, HD-site64_1587589966, N41site65_1587590071, 3B_1587589656, SS-NP203_1587590145, FC-NESR1_1587590219, NESSE_1587597146, BICY_1587504468, SR-gun_1587589447. https://sofia.usgs.gov/eden/stationlist.php). (TIF) [file pone.0326148.s008.tif]

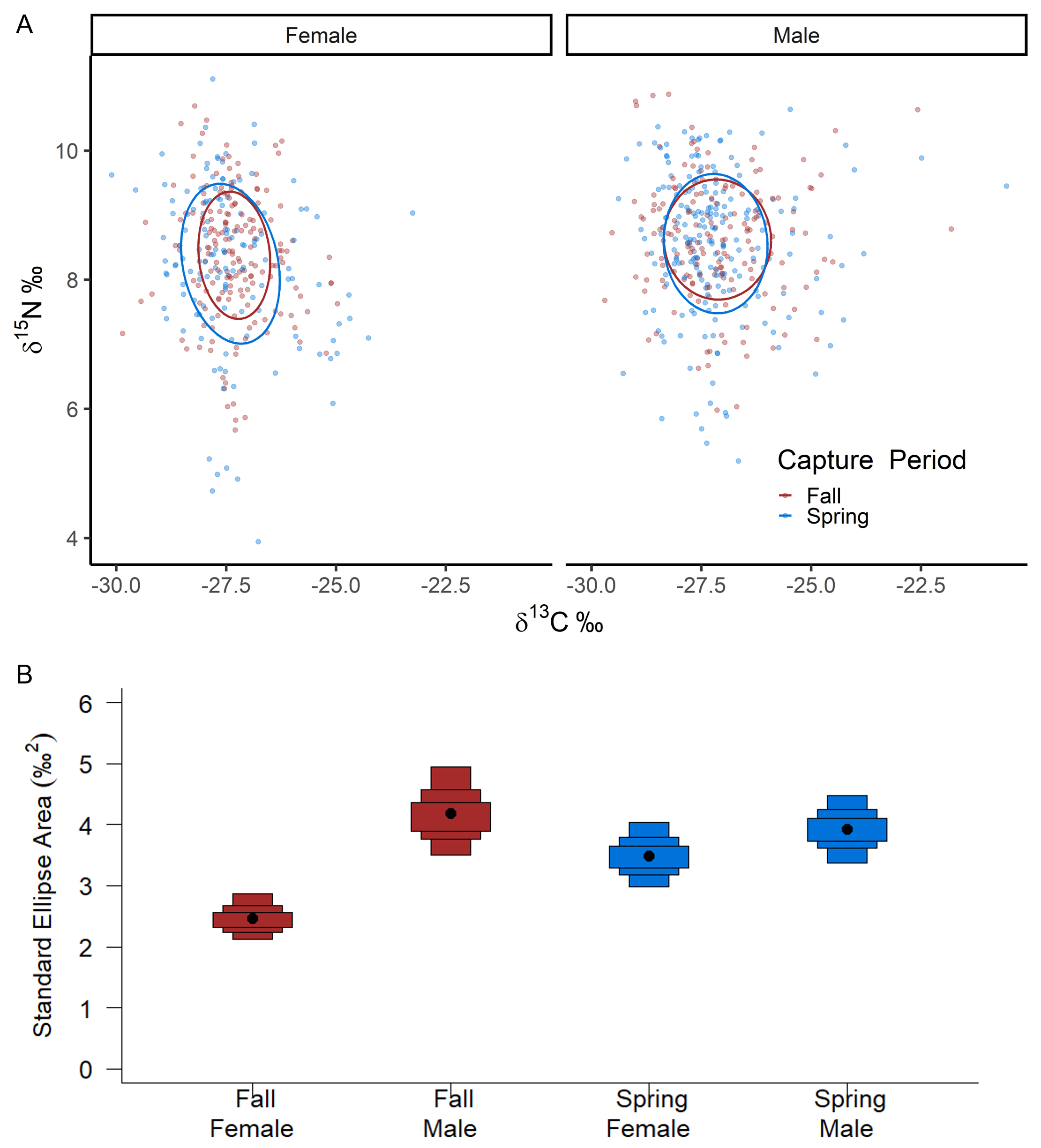

Supplement: S8 Fig — Estimated 40% core isotopic niches for each capture period (season) by A) Sex; blue circles and ellipses represent male alligators and red circles and ellipses represent females, B) Bayesian estimated niche area SEAB for each sex and capture period. Boxes represent 50%, 75%, and 95% credibility intervals, and black dots correspond to the median. (TIF) [file pone.0326148.s009.tif]

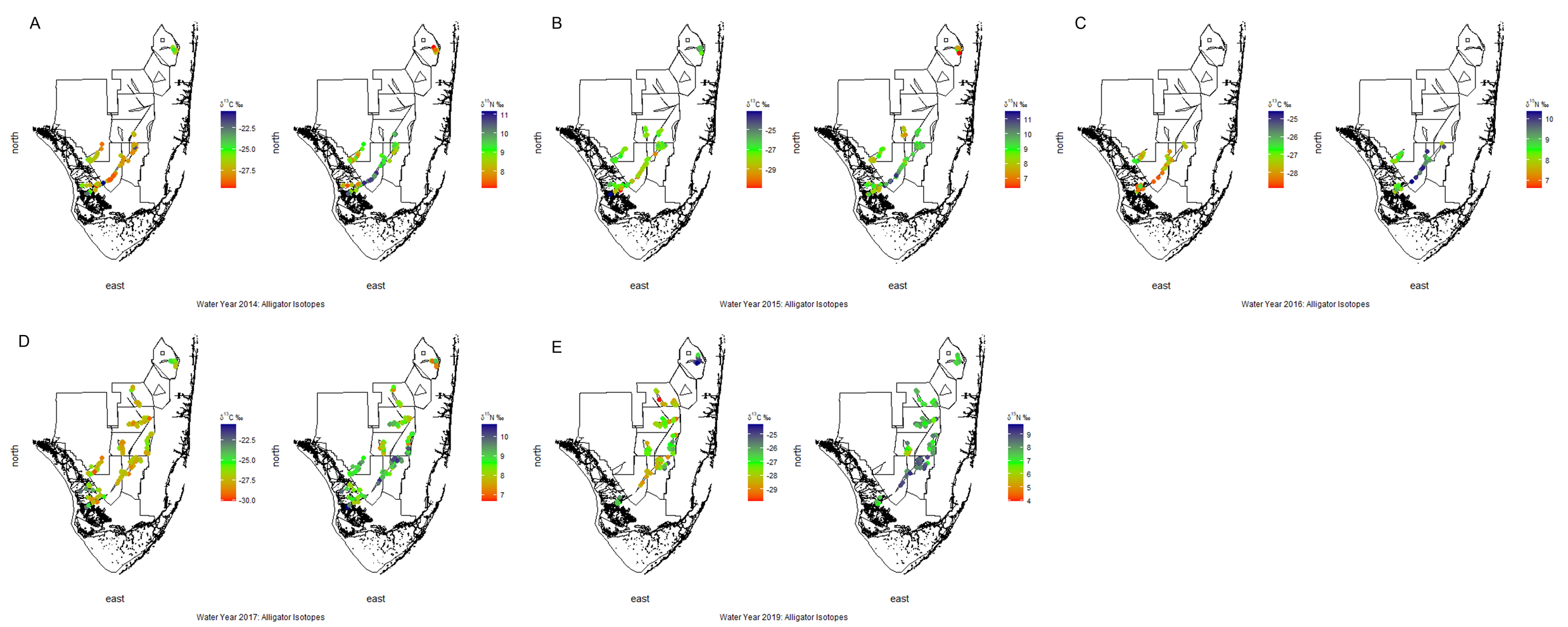

Supplement: S9 Fig — Capture locations of American alligator (Alligator mississippiensis) within the southern Everglades ecosystem, Florida, USA depicted by isotopic mean δ13C (left) and δ15N (right) values by water year A) 2014, B) 2015, C) 2016, D) 2017, E) 2019 *Note, not all sites were sampled each water year. (TIF) [file pone.0326148.s010.tif]

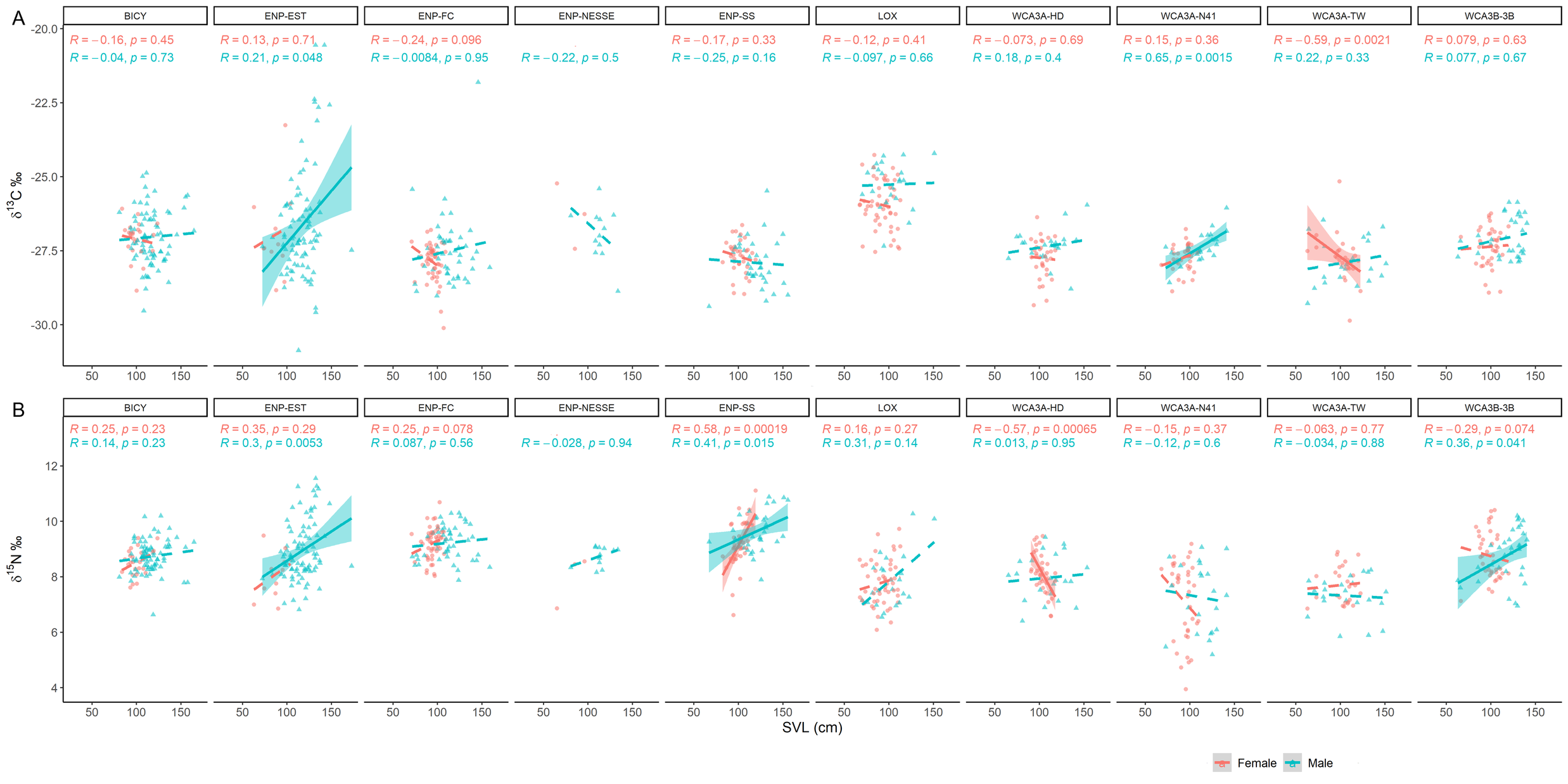

Supplement: S10 Fig — Relationships of (A) δ13C and (B) δ15N with the sex-SVL interaction from 688 capture event tissue samples from 647 American alligators within the southern Everglades ecosystem, Florida, USA during this study (*Excludes 5 individuals where sex was not determined). The ten sampling sites were within five wetland watersheds, Arthur R. Marshall Loxahatchee National Wildlife Refuge (LOX), Big Cypress National Preserve (BICY), estuaries within southwestern Everglades National Park (ENP-EST), and multiple sites within Water Conservation Area 3 (WCA3; WCA3A-TW (Tower), WCA3A-HD (Holiday Park), WCA3A-N41, WCA3B-3B), and Everglades National Park freshwater marshes (ENP-FW; ENP-FC (Frog City), ENP-SS (Shark Slough), ENP-NESSE (NE Shark Slough). (TIF) [file pone.0326148.s011.tif]

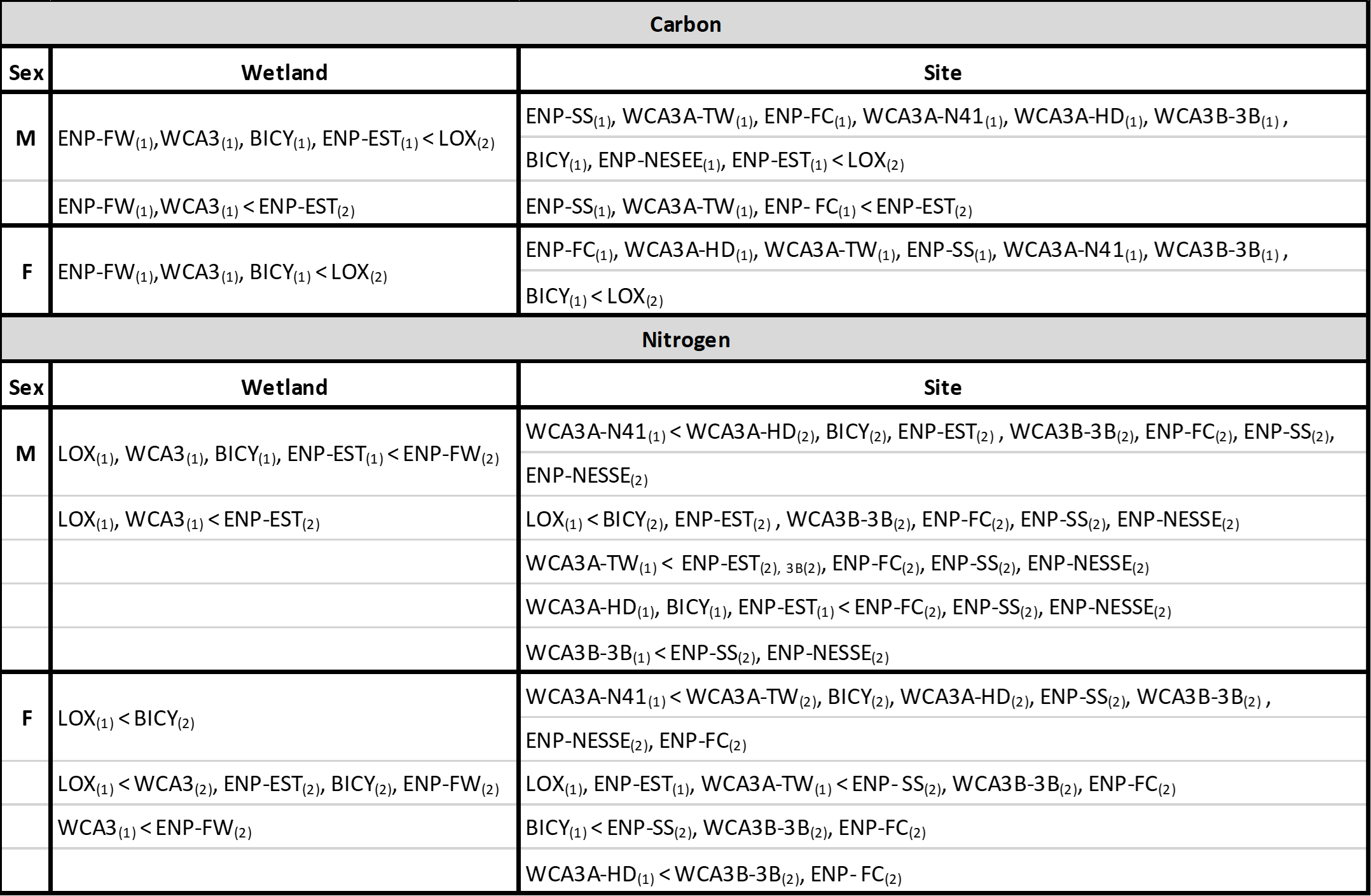

Supplement: S1 Table — *Significant differences between sites are represented by dissimilar subscripts. (TIF) [file pone.0326148.s012.tif]

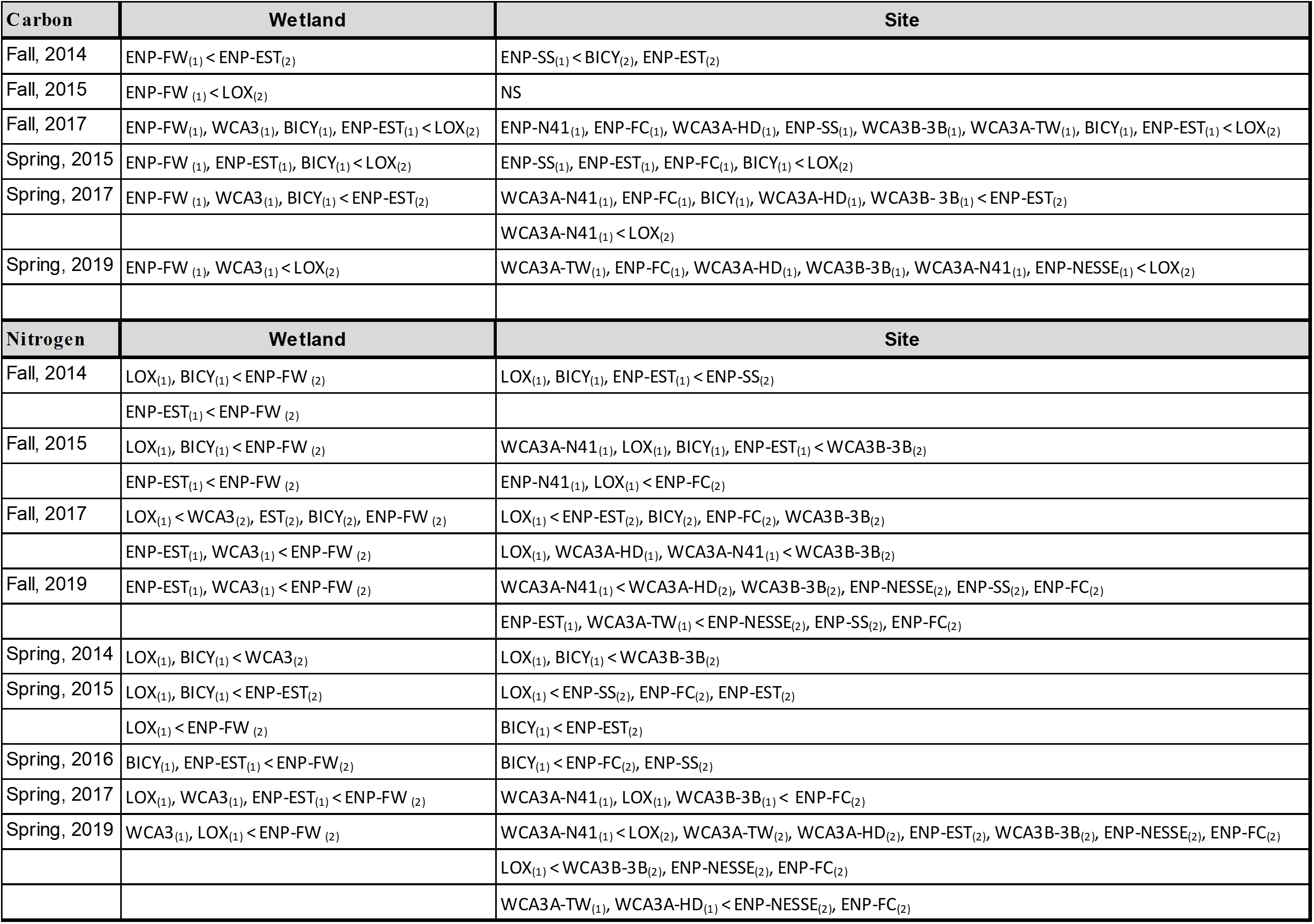

Supplement: S2 Table — *Significant differences between sites are represented by dissimilar subscripts. (TIF) [file pone.0326148.s013.tif]

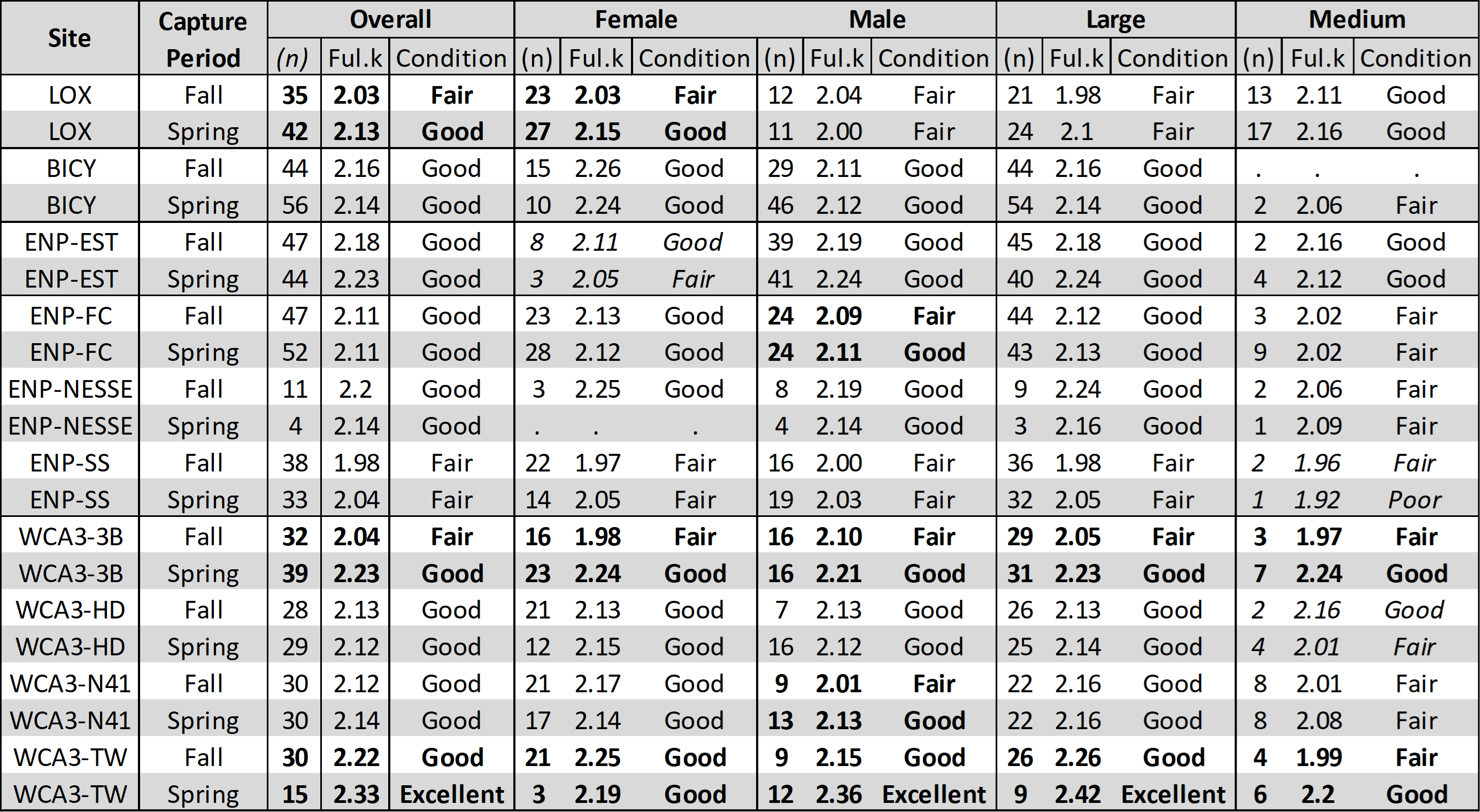

Supplement: S3 Table — Fulton’s K equation used to generate body condition values [K = M/SVL3 × 105], classified as Poor (≤1.95), Fair (>1.95– ≤ 2.1), Good (>2.1– ≤ 2.27), or Excellent (>2.27). Body condition values in bold were higher in spring capture period, those in italic were higher in fall capture periods, normal font no difference between capture periods. *Excludes alligators in the small size class and those whose sex was undetermined. (TIF) [file pone.0326148.s014.tif]
